# *Bacillus subtilis* serine/threonine protein kinase YabT is involved in spore development via phosphorylation of a bacterial recombinase

**DOI:** 10.1111/mmi.12233

**Published:** 2013-05-02

**Authors:** Vladimir Bidnenko, Lei Shi, Ahasanul Kobir, Magali Ventroux, Nathalie Pigeonneau, Céline Henry, Alain Trubuil, Marie-Françoise Noirot-Gros, Ivan Mijakovic

**Affiliations:** 1 INRA, UMR1319 MicalisJouy-en-Josas, F-78350, France; 2 AgroParisTech, UMR1319 MicalisJouy-en-Josas, F-78350, France; 3 Unité de recherche Mathématiques et Informatique Appliquées, INRAJouy-en-Josas, FR-78350, France

## Abstract

We characterized YabT, a serine/threonine kinase of the Hanks family, from *Bacillus subtilis*. YabT is a putative transmembrane kinase that lacks the canonical extracellular signal receptor domain. We demonstrate that YabT possesses a DNA-binding motif essential for its activation. *In vivo* YabT is expressed during sporulation and localizes to the asymmetric septum. Cells devoid of YabT sporulate more slowly and exhibit reduced resistance to DNA damage during sporulation. We established that YabT phosphorylates DNA-recombinase RecA at the residue serine 2. A non-phosphorylatable mutant of RecA exhibits the same phenotype as the Δ*yabT* mutant, and a phosphomimetic mutant of RecA complements Δ*yabT*, suggesting that YabT acts via RecA phosphorylation *in vivo*. During spore development, phosphorylation facilitates the formation of transient and mobile RecA foci that exhibit a scanning-like movement associated to the nucleoid in the mother cell. In some cells these foci persist at the end of spore development. We show that persistent RecA foci, which presumably coincide with irreparable lesions, are mutually exclusive with the completion of spore morphogenesis. Our results highlight similarities between the bacterial serine/threonine kinase YabT and eukaryal kinases C-Abl and Mec1, which are also activated by DNA, and phosphorylate proteins involved in DNA damage repair.

## Introduction

Protein phosphorylation is a wide-spread post-translational modification that allows rapid and reversible regulation of protein activity (Cohen, 2000). Bacteria and Eukarya share the same super-family of Hanks-type protein kinases (Hanks *et al*., 1988; Leonard *et al*., 1998) capable of phosphorylating proteins on serine/threonine and tyrosine in Eukarya, and only on serine/threonine in bacteria (Pereira *et al*., 2011). Canonical Hanks-type kinases are transmembrane proteins. Their highly conserved kinase domain is cytosolic, and a transmembrane helix connects it to the extracellular domain. The structure of the extracellular domain varies considerably, presumably to accommodate various ligands that constitute activation signals for the kinase (Pereira *et al*., 2011). The activation of Hanks-type kinases involves ligand binding to the extracellular domain, which usually provokes dimerization, and trans-autophosphorylation of the kinase ‘activation loop’, thus rendering the kinase fully active (Greenstein *et al*., 2007). The distribution of Hanks-type kinases in bacteria is quite uneven (Wehenkel *et al*., 2008). Some bacteria contain no kinases belonging to this super-family, whereas species of *Actinomycetales*, *Myxococcales*, *Nostocales* are particularly rich in Hanks-type kinases (Perez *et al*., 2008). Among the 130 sequenced genomes of Firmicutes, 123 possess genes for Hanks-type kinases. The genome of the Firmicute model organism *B. subtilis* is known to encode three Hanks-type kinases: PrkC, PrkD and YabT. Two of them, PrkC and PrkD, have characterized physiological substrates. PrkC exhibits a canonical Hanks-type kinase structure, and was first characterized as a transition/stationary phase kinase that phosphorylates the elongation factor G (Gaidenko *et al*., 2002). Identification of other substrates of PrkC followed soon thereafter (Absalon *et al*., 2009). Next, the PrkC activation mechanism was described: it is activated by binding muropeptides (Shah *et al*., 2008), and it phosphorylates ribosomal and ribosome-associated proteins (Shah *et al*., 2008; Pompeo *et al*., 2012) as well as glycolytic enzymes (Pietack *et al*., 2010). PrkC activation by muropeptides and subsequent substrate phosphorylation has been shown to contribute to spore germination (Shah *et al*., 2008). PrkD (also known as YbdM) is a soluble cytosolic protein. It was found to phosphorylate a two-component kinase DegS, and activate phosphotransfer from DegS to DegU (Jers *et al*., 2011). This influences the expression from promoters involved in competence, swarming and complex colony formation, which are all co-regulated by P∼DegU (Jers *et al*., 2011). The signal activating PrkD is not yet known. Studies of serine/threonine phosphorylation in *B. subtilis* are facilitated by existing phosphoproteome analyses for this model organism, which include several site-specific studies (Macek *et al*., 2007; Soufi *et al*., 2010). Currently, in the list of known *B. subtilis* phosphoproteins, there are over 70 serine/threonine phosphorylation sites without an assigned kinase.

In this study we set out to characterize the remaining Hanks-type kinase of *B. subtilis*, YabT, which had no assigned physiological role or substrates. We show that YabT expression profile has a peak in the early hours of spore development, and *in vivo* YabT localizes to the septum between the mother cell and the nascent spore. YabT possesses a putative transmembrane helix, but has no extracellular signal-binding domain. Surprisingly, we found that this kinase is activated by binding DNA. Inactivation of the *yabT* gene *in vivo* leads to increased sensitivity to DNA damage during sporulation. Based on these findings, we speculated that YabT might play a role similar to eukaryal kinases C-Abl and Mec1, which are activated by DNA-damage and phosphorylate proteins involved in damage repair (Yuan *et al*., 1998; Herzberg *et al*., 2006; Shimizu *et al*., 2009; Flott *et al*., 2011). We identified a substrate of YabT, which is the general DNA-recombinase RecA, involved in homologous recombination and DNA damage repair (Lusetti and Cox, 2002). DNA recombinases participate in homologous recombination and recombinational repair of DNA double-strand breaks in all living forms (Kuzminov, 2001). They are ssDNA-binding ATPases that promote homologous DNA strand exchange through formation of a dynamic nucleoprotein filament (Chen *et al*., 2008). Bacterial RecA proteins also participate in initiation of the cellular SOS response to DNA damage, by acting as co-proteases for the LexA repressor (Butala *et al*., 2009). While these roles of RecA have been extensively studied in exponentially growing bacteria, precious little is known about RecA role during sporulation. Inactivation of *recA* has been shown to reduce sporulation efficiency of *B. subtilis* (Shafikhani *et al*., 2004), and it provoked a defect in prespore nucleoid condensation (Sciochetti *et al*., 2001). We demonstrate that the non-phosphorylatable mutant of RecA exhibits the same phenotype as the Δ*yabT* strain, and the phosphomimetic mutant of RecA can restore the phenotype of Δ*yabT* into wild type. We show that phosphorylation promotes the formation of transient RecA foci, and if these foci persist at later stages of spore development, they become incompatible with fully developed spores.

## Results and discussion

### YabT kinase is activated by DNA-binding

**Fig. 1:**
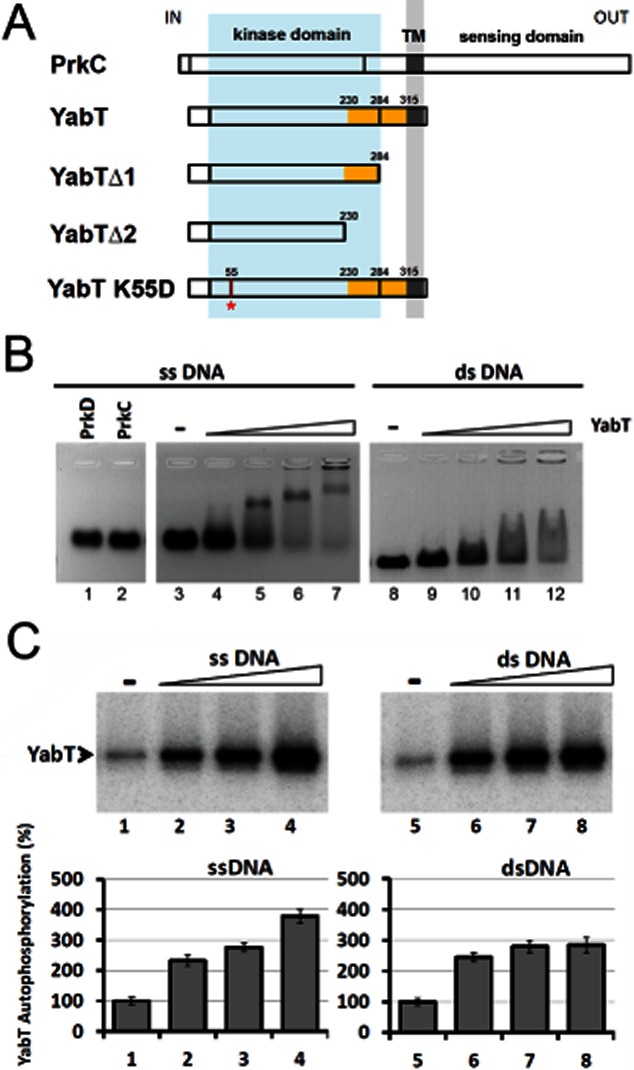
YabT is activated by DNA binding. A. Schematized structure of YabT aligned to reference Hanks kinase PrkC, showing the kinase domain (blue shading) and the transmembrane helix (dark grey). Truncated versions YabTΔ1 and YabTΔ2 are shown, with the putative DNA-binding region (residues 230–315) highlighted in orange. Catalytic mutant YabT K55A has the mutated residue K55 highlighted in red. B. Binding of YabT to random sequence ssDNA (140 bases fragment, 0.4 μM) and dsDNA (210 base pairs fragment, 0.25 μM), in a gel-shift assay. *B. subtilis* kinases PrkD and PrkC were included (4.5 μM each) as controls in lanes 1 and 2, YabT concentrations are 0, 2.0, 3.0, 4.0, 5.0 μM (lanes 3–7) and 0, 1.5, 3.0, 4.5, 6.0 μM (lanes 8–12). C. Autoradiography of SDS-polyacrylamide gels showing the influence of DNA binding on YabT autophosphorylation. 0.25 μM YabT was incubated for 2 h with 0, 3, 6, 12 nM of random-sequence ssDNA (140 bases fragment) in lanes 1 to 4, and 0, 3, 6, 12 nM of dsDNA (210 base-pairs fragment) in lanes 4 to 8, in the *in vitro* phosphorylation assay. Quantification was performed on results from three independent experiments and the representative gels are shown. Columns 1 to 8 correspond to lanes 1 to 8 in the gels respectively. Signals were normalized with respect to autophosphorylation of YabT incubated without DNA (lanes 1 and 5) that was taken as 100%.

The *B. subtilis* Hanks-type kinase PrkC (Gaidenko *et al*., 2002) exhibits a structure that is typical for proteins of its family (Pereira *et al*., 2011) (Fig. 1A). Its catalytic kinase domain is at the N-terminus, it is followed by a transmembrane helix, connecting to an extracellular domain responsible for ligand binding and activation (Shah *et al*., 2008). By comparison, YabT architecture is unusual for a Hanks-type kinase (Fig. 1A). YabT possesses the catalytic domain followed by a putative transmembrane helix, but lacks the external sensing domain. This suggests that the activating signal for YabT might come from the cytosol. In the cytosolic part of YabT, partially overlapping with the catalytic domain, there is a region rich in lysine and arginine residues (marked in orange, Fig. 1A), stretching from residues 230–315. This region is absent in YabT paralogues, and most of its orthologues in other bacteria. Since regions with positively charged residues characterize DNA-binding proteins, and some eukaryal Hanks-type kinases are known to bind DNA (Shimizu *et al*., 2009; Flott *et al*., 2011), we tested the ability of YabT to bind DNA *in vitro*. We set up a DNA-binding assay using random sequence double stranded (ds) and single stranded (ss) DNA, purified YabT, and purified PrkC and PrkD as controls. Unlike any other characterized bacterial Hanks-type kinase, YabT was capable of binding both ssDNA and dsDNA (Fig. 1B). In our experimental setup, binding of ssDNA seemed to be more efficient. PrkC and PrkD, devoid of the region containing positively charged residues, did not shift DNA. YabT binding of DNA was not sequence specific, and could be achieved with DNA fragments with minimal length of 15 bases (data not shown). Our *in vitro* autophosphorylation assay revealed that YabT can autophosphorylate in the absence of DNA, but the presence of either dsDNA or ssDNA enhanced the phosphorylation activity of YabT significantly (Fig. 1C). Again, ssDNA was more efficient in activating YabT. While activation with dsDNA reached saturation at around 6 nM, activation with ssDNA progressed linearly between 6 and 12 nM DNA. We therefore speculated that DNA, and more particularly ssDNA, could constitute the activation signal for YabT.

### Residues 230–315 are responsible for YabT activation via DNA binding

**Fig. 2:**
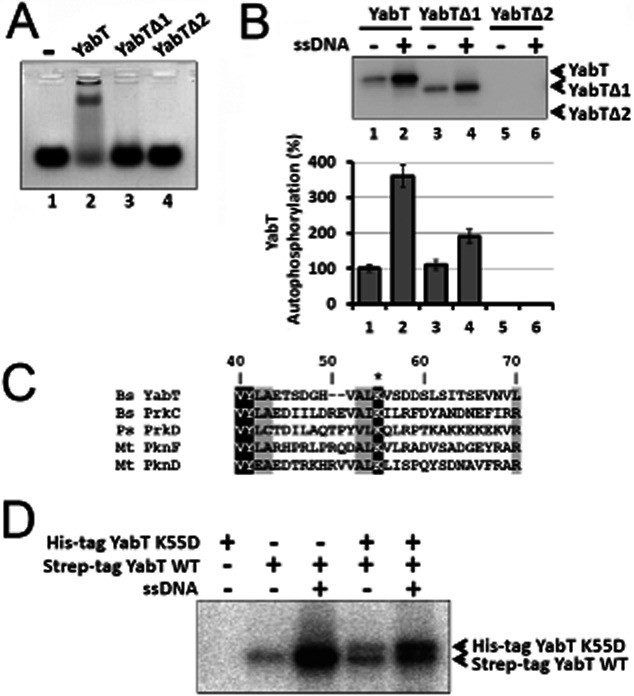
YabT DNA binding site and trans-autophosphorylation. A. Binding of YabTΔ1 and YabTΔ2 to 0.4 μM 140 bases ssDNA fragment, in a gel-shift assay. No YabT was added in lane 1 as control, and 4 μM of each protein were in lanes 2 to 4. B. *In vitro* autophosphorylation assay of YabT, YabTΔ1 and YabTΔ2. 0.25 μM YabT, YabTΔ1 and YabTΔ2 were incubated in the presence of 12 nM ssDNA fragment (lanes 2, 4 and 6), and in the absence of ssDNA fragment (lanes 1, 3 and 5). Quantification was performed from three independent experiments and the representative gel is shown. All data were normalized with respect to the autophosphorylation signal of YabT incubated without DNA (lane 1) that was defined as 100%. Reactions were incubated for 2 h before separation on SDS-PAGE. C. ClustalW alignment showing the conservation of the catalytic YabT residue K55. Other bacterial Hanks kinases shown are PrkC and PrkD from *B. subtilis* and PknD and PknF from *Mycobacterium tuberculosis*. D. *In vitro* phosphorylation assay showing trans-autophosphorylation reaction between YabT wild type and YabT K55D. The presence or absence of key reactants (12 nM 140 nt ssDNA, 0.08 μM YabT WT and 0.16 μM YabT K55D) is indicated as +/− above each lane. Reactions were incubated for 2 h before separation on SDS-PAGE.

To confirm our hypothesis that the YabT region between residues 230–315 is responsible for DNA-binding, we constructed two deletions: YabTΔ1 and YabTΔ2 (Fig. 1A). YabTΔ1 contains residues 1–284, and thus preserves the entire kinase domain, but it misses the positively-charged residues 285–315. YabTΔ2 contains the residues 1–230, thus being devoid of the entire region of positively charged residues, but it also inevitably lacks a part of the kinase domain (residues 231–284). As can be seen in the binding assay (Fig. 2A), YabTΔ1 was severely impaired in DNA binding, and YabTΔ2 has lost the ability to bind DNA, confirming that the entire region 230–315 is required for DNA binding. YabTΔ2 has also lost all autokinase activity, irrespective of presence of DNA (Fig. 2B), which was expected since residues 231–284 constitute an essential part of the kinase domain. The protein YabTΔ1 retained almost wild-type level of kinase activity in the absence of DNA, but could not be fully activated in the presence of DNA (Fig. 2B). This result suggests that the lysine/arginine rich region of YabT between residues 284–315 is sufficient to bind DNA and activate the kinase function of YabT. Upon ligand binding, Hanks-type kinases usually dimerize and one interaction partner phosphorylates the activation loop of the other interaction partner in an inter-molecular event called trans-autophosphorylation (Greenstein *et al*., 2007; Pereira *et al*., 2011). Dimerization and trans-autophosphorylation have been characterized in detail for *B. subtilis* PrkC, which autophosphorylates 4 threonines in the activation loop situated between residues 162–167 (Madec *et al*., 2003).

In order to test whether DNA binding of YabT could promote trans-autophosphorylation, we needed an active site mutant devoid of kinase activity. The active site of Hanks-type kinases is highly conserved, and the degree of homology allows for homology-based structural modelling. Therefore, we used the SWISS-MODEL Workspace (Arnold *et al*., 2006) to create such models of YabT. The models (data not shown) and the underlying alignments clearly identified lysine 55 as the catalytic residue, involved in the hydrolysis of ATP bound in the active site (Fig. 2C). We thus constructed a point-mutated version of YabT, YabT K55D, replacing the catalytic lysine 55 with aspartate. This protein was unable to autophosphorylate in our *in vitro* phosphorylation assay (Fig. 2D). Next, we produced a strep-tagged version of wild type YabT in order to distinguish it from 6x-His tagged YabT K55D. As presented in Fig. 2D, YabT was able to trans-phosphorylate YabT K55D irrespective of the presence of DNA, although both autophosphorylation and trans-phosphorylation signals were increased in the presence of DNA. Our interpretation of these findings is that YabT is capable of trans-molecular autophosphorylation like its homologues, but this reaction does not require DNA. YabT can be activated by DNA fragments as short as 15 bases, which corresponds to approximately 5.5 nm in length (Adamcik *et al*., 2006). Since the diameter of a Hanks-type kinase catalytic domain is approximately 3.5 nm (Young *et al*., 2003), it is unlikely that this fragment can span two domains. Therefore we conclude that the activation by DNA is most probably an intra-molecular event.

### YabT is expressed during sporulation and enriched at the septum between the mother cell and forespore

**Fig. 3:**
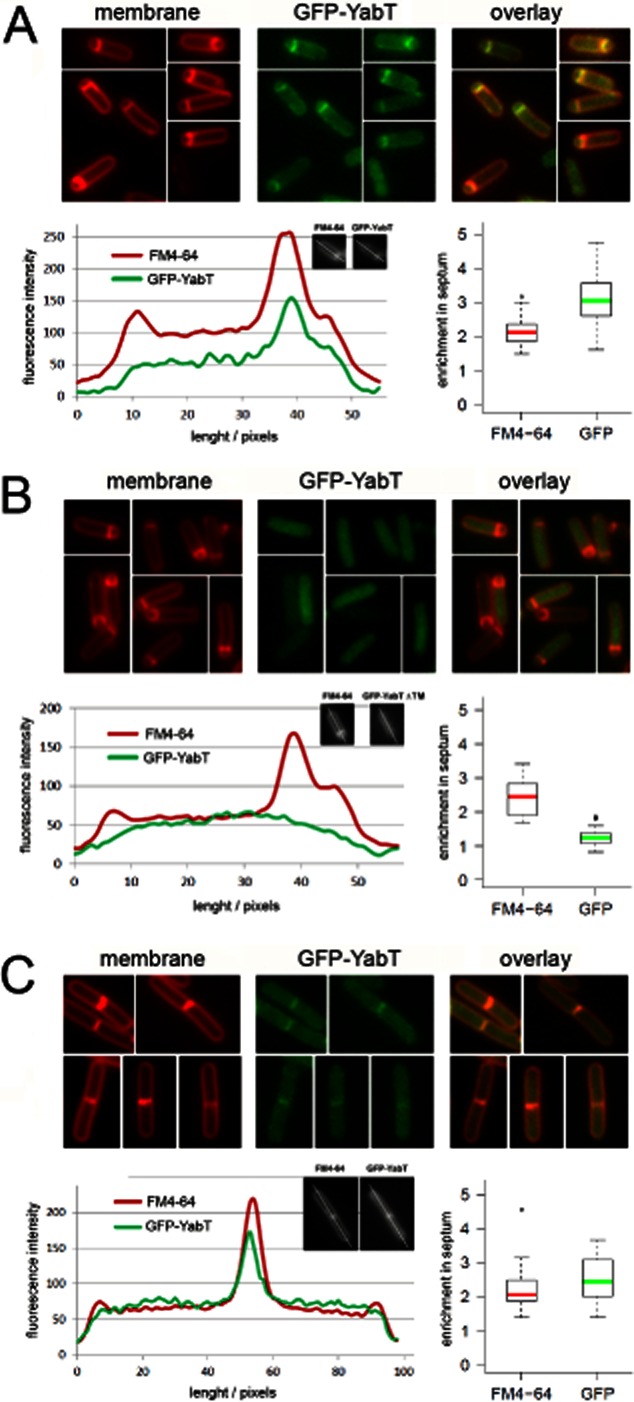
YabT enriched at the septum during sporulation. A–C. Fluorescence microscopy of *B. subtilis* cells expressing a xylose-inducible GFP-YabT fusion. For the images at the top of each panel, red fluorescence indicates cell membranes stained with FM4-64 (left), green fluorescence indicates localization of GFP-YabT (middle), and they are also shown in overlay (right). Quantification of fluorescence along the cell axis is shown for a typical cell in the bottom left part of each panel. Based on this quantification for all examined cells, an enrichment profile at the septum has been calculated for FM4-64 and GFP-YabT fluorescence, bottom right of each panel. The enrichment is calculated as the ratio of fluorescence at the septum divided by the fluorescence at the opposite polar peak for sporulating cells, and average value of both polar peaks for exponential cells. A. Localization of GFP-YabT during sporulation, images taken at T3. B. Localization of GFP-YabTΔTM during sporulation, images taken at T3. C. Localization of GFP-YabT expressed during exponential growth.

So far we have established that YabT is a protein kinase activated by binding DNA. In order to explore when and where YabT encounters DNA in the cell we shifted our attention to *in vivo* assays. YabT-encoding gene was previously reported to be expressed in a SigF-dependent manner (Wang *et al*., 2006). SigF is a special sigma factor dedicated to the regulation of spore development in *B. subtilis* (Haldenwang, 1995). When exposed to lack of nutrients, the ultimate stationary phase response of *B. subtilis* is the morphological differentiation of a highly resistant life form called the spore. The spore preserves the chromosome in a resistant dormant cell, which germinates into a vegetative bacterium once the favourable conditions return (Errington, 2003). Spore development is ensured by a tightly regulated spatio-temporal program which co-ordinates the sequential activation of hundreds of specific genes (Fujita and Losick, 2005). Throughout this manuscript we will designate the time-point of initiation of spore development as the time T0. Subsequent time-points will be designated as T1 for one hour after T0, T2 for two hours after T0, etc. Fig. S1 presents the transcription profile of the *yabT* gene, compared with that of *prkC* and *prkD* (Nicolas *et al*., 2012). As expected from its published physiological role, *prkC* expression is boosted during spore germination (Shah *et al*., 2008). By contrast, the transcription of *yabT* peaks out 3 h after the onset of sporulation. This is consistent with *yabT* being controlled by SigF, whose targets are known to reach maximal expression between T2.5 and T4.5 (Haldenwang, 1995). Due to post-transcriptional and post-translational control, it is possible that the actual protein levels differ from the mRNA levels. Therefore we wanted to check directly the YabT protein levels *in vivo*. We introduced a SPA-tagged version of *yabT* at the locus, under control of its native promoter. The expression of YabT-SPA was followed between T0 and T5 by Western blot, using anti-SPA antibodies (Fig. S1). YabT-SPA protein was not detectable before T2, and the maximal levels were reached at T4. Western and transcriptome-derived profiles of expression were thus in agreement, with a major peak of *yabT* expression during spore development, reaching a maximum between T3 and T4. Next, to pin-point the sub-cellular localization of YabT, we fused it with the green fluorescent protein (GFP). YabT-GFP expressed from the locus did not provide enough fluorescence at time T3 to establish a localization profile. Moreover, C-terminal location of GFP, i.e. close to the putative transmembrane domain of YabT might impede its proper localization. For these reasons, we used N-terminal GFP-YabT fusion synthesized from a xylose-inducible promoter. We have shown that this fusion is functional as a kinase *in vitro*, and can complement the Δ*yabT* phenotype (mentioned in the next paragraph) *in vivo* (Fig. S2). When GFP-YabT was analysed at the time point T3 (maximum of *yabT* expression), GFP-YabT colocalized strongly with the septal membrane separating the mother cells from the forespore (Fig. 3A). 67% of examined cells exhibited septa, and in 66% of those GFP-YabT presented a septal localization profile. A representative sample of these is shown in the top of the panel (Fig. 3A). At the septum, the membrane fluorescence was enriched by a factor of 2, whereas GFP-YabT signal was enriched about threefold (Fig. 3A, bottom right). A statistical comparison (*T*-test) of the red and green fluorescence enrichment profiles yielded the *P*-value of 7.5 × 10^−9^, indicating that the two are significantly different. This suggests that YabT localization is specifically enriched at the septum at T3. Interestingly, deletion of the YabT putative transmembrane domain from the GFP-YabT fusion provoked a loss of septal localization (Fig. 3B). Among examined cells 61% contained septa, and not a single one exhibited YabT septal localization. Accordingly, the enrichment ratio of the green fluorescence at the septum was close to 1 for this strain (Fig. 3B, bottom right), significantly lower than the FM4-64 signal (*P*-value of 2.6 × 10^−10^). In order to assess whether septal localization of YabT is sporulation-specific, we expressed the GFP-YabT in exponential phase (Fig. 3C). In these conditions, 39% of examined cells exhibited a symmetrical division septum. 91% of these presented some enrichment of GFP-YabT at the septum, but the enrichment did not significantly exceed the twofold ratio exhibited by the FM4-64 signal (*P*-value of 0.14). This suggests that septal localization of YabT is growth stage specific, its enrichment at the septum occurs specifically during sporulation, and requires the putative transmembrane domain of YabT. During spore development, before the septum is sealed, one copy of the chromosome has to go through and enter the spore. There it can encounter YabT, and this encounter can presumably activate the YabT kinase function.

### Loss of YabT leads to increased sensitivity to DNA damage during spore development

**Fig. 4:**
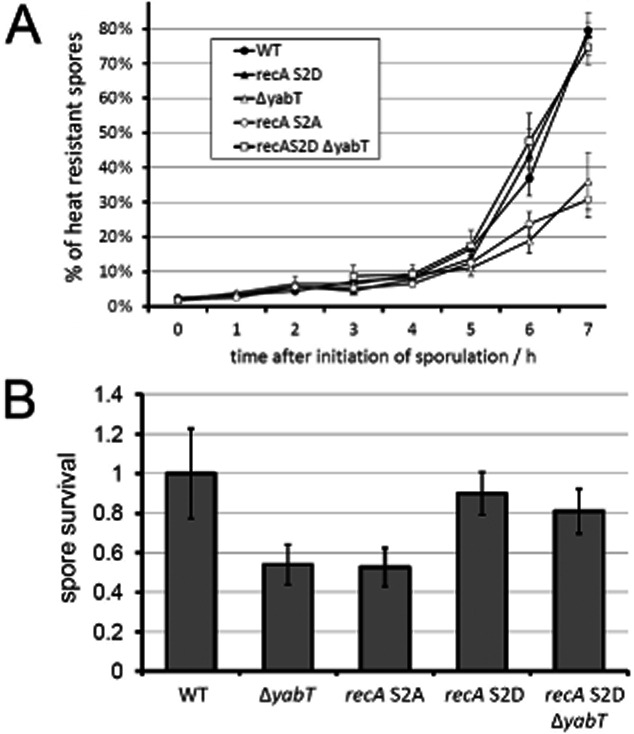
Phenotypes of Δ*yabT* and *recA* point-mutants during sporulation. A. Kinetics of spore formation of wild type *B. subtilis* and strains *recA* S2A, *recA* S2D, Δ*yabT* and *recA* S2D Δ*yabT* in the sporulation medium from stage T0 to T7. Spore counts are expressed as % of total number of viable cells. Error bars represent standard deviation from 5 biological replicates. B. Spore survival after mitomycin treatment (20 ng ml^−1^) applied at time-point T1 to the sporulating cultures of *B. subtilis* strains *recA* S2A, *recA* S2D, Δ*yabT*, *recA* S2D Δ*yabT* and wild type. Spore counts are expressed as number of spores in treated culture/number of spores in untreated culture for each strain, normalized with respect to the wild type. Error bars represent standard deviation from 5 biological replicates.

The expression and localization data describe YabT as a kinase that is specifically expressed at T2-T4 during spore development and localizes to the septum. It is therefore plausible to presume that YabT might be involved in sporulation. To test this assumption, we inactivated the *yabT* gene. First, we examined the kinetics of sporulation of the Δ*yabT* strain, using an assay that counts heat-resistant spores and expresses them as the fraction of total viable cells (Fig. 4A). Cells devoid of YabT were clearly slower to sporulate than the wild type. In Δ*yabT* strain only 35% of cells were heat resistant (converted to spores) after 7 h, while in the wild type the spore count reached 80% in the same interval. After 24 h, Δ*yabT* reached approximately the same total level of spores as the wild type (data not shown) indicating that the difference is only in time-course, but not in the overall capacity to develop mature spores. At this stage, looking for a phenotype of Δ*yabT*, we were prompted to draw parallels with eukaryal kinases that are DNA-activated. For example, C-Abl is activated by associating with DNA, and as a consequence phosphorylates the recombinase Rad51 (Shimizu *et al*., 2009). C-Abl is activated by DNA damage and influences the participation of Rad51 in damage repair (Yuan *et al*., 1998). Another DNA damage-responsive kinase, Mec1, phosphorylates Rad51 at serine 192, directly affecting its ATPase function, and subsequently its role in damage repair (Flott *et al*., 2011). In the light of these results, we decided to add the DNA-damaging agent mitomycin to sporulating cells at T1, and detect spore survival. Spore survival for each strain was expressed as the ratio of heat-resistant spore counts from mitomycin-treated and non-treated cultures (Fig. 4B). The Δ*yabT* strain spores exhibited about 50% less survival compared with the wild type under DNA damaging conditions. This indicated that the role of YabT during sporulation might indeed be connected to DNA damage.

### YabT phosphorylates a DNA recombinase RecA

We speculated that the phenotype of Δ*yabT* strain might be related to YabT-dependent phosphorylation of some cellular protein substrate. As argued in the introduction, ssDNA-activated kinases in Eukarya phosphorylate proteins involved in DNA damage repair. In *B. subtilis* the recombinase RecA was found to be phosphorylated *in vivo* at the residue serine 2 (Soufi *et al*., 2010) and arginine 58 (Elsholz *et al*., 2012).

**Fig. 5:**
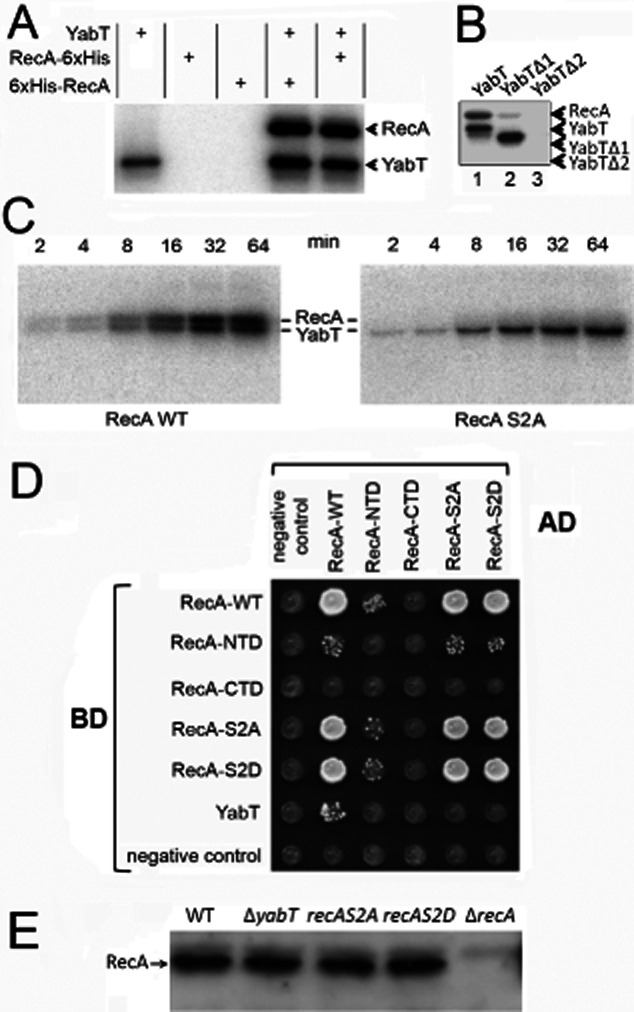
Phosphorylation of RecA by YabT. A. Autoradiography of SDS-polyacrylamide gels showing *in vitro* phosphorylation assays with YabT and RecA fused with an N-terminal His_6_-tag (6xHis-RecA) or C-terminal His_6_-tag (RecA-6xHis). Experiment was performed with 0.25 μM YabT and 1 μM RecA (either N- or C-terminally tagged). Presence of key proteins in the assays is indicated with +/− above each lane. Reactions were incubated for 2 h prior to separation by SDS-PAGE. Bands corresponding to autophosphorylated YabT and phosphorylated RecA are indicated by arrows. B. In lanes 1–3, phosphorylation of RecA by three versions of YabT was examined. 1 μM RecA was incubated with 0.25 μM YabT, YabTΔ1 and YabTΔ2. Reactions were incubated for 2 h before separation on SDS-PAGE. C. Autoradiography of SDS-polyacrylamide gels showing *in vitro* YabT-dependent phosphorylation of RecA wild type and RecA S2A. Time dependence of the reaction was followed and the incubation times are given above each lane. Bands corresponding to autophosphorylated YabT and phosphorylated RecA are indicated. D. Yeast two-hybrid phenotypic interaction assay with YabT and RecA (wild type, truncated and mutant derivatives). Yeast haploid cells expressing the YabT protein, the RecA full sized, or N and C-terminal domains (NTD and CTD respectively), as well as the S2A and S2D mutant derivatives were fused with the binding domain of Gal4 (BD) and mated against compatible haploid cells expressing the matching RecA derivatives fused with the activating domain (AD). The interacting phenotypes were monitored by the ability of mating products to grow on the selective medium -LUH. E. RecA levels in different strains were probed by Western blot using anti-RecA antibodies on crude extracts obtained at T3. All protein extracts were normalized to the same total protein concentration using a Bradford assay, and equal amount of total protein was loaded in each lane. Strains are indicated above each lane, and the band corresponding to RecA is indicated by an arrow.

We first wanted to test if RecA could be a substrate for YabT dependent phosphorylation *in vitro*. For this purpose we purified RecA with a His-tag. We placed the tag at either its N- or C-terminus, since the phosphorylated residue S2 is close to the N-terminus and we wanted to exclude the possibility that a tag positioned close to the phosphorylation site could cause an artifactual result. Our *in vitro* phosphorylation assay revealed that neither of the two tagged versions of RecA could autophosphorylate, and both were phosphorylated in the presence of YabT, with a comparable efficiency (Fig. 5A). Thus we established that YabT could phosphorylate RecA *in vitro*. Interestingly, truncated YabTΔ1 retained some residual ability to phosphorylate RecA, whereas YabTΔ2 could not phosphorylate the substrate at all, due to loss of a part of its catalytic domain (Fig. 5B). Our next question was whether this phosphorylation is specific for the residue serine 2, found to be phosphorylated *in vivo*. To test this, we constructed a point mutant of RecA replacing the serine 2 with alanine, a residue that cannot be phosphorylated. Then we compared the efficiency of YabT-dependent phosphorylation *in vitro* for RecA S2A and RecA wild type (Fig. 5C). YabT-dependent phosphorylation was completely abolished in RecA S2A, indicating that the kinase is indeed specific for this residue. To confirm the interaction between YabT and RecA in a context more closely related to *in vivo* settings, we performed a yeast two-hybrid assay with various forms and mutants of RecA used as both bait and prey (for control), and tested for their interaction with wild type YabT (Fig. 5D). We found that the ability of RecA to self-interact was not affected by the presence of the Gal4 N-terminally fused domains, but required RecA integrity as it was abolished or weakened by deletions of either C- or N-terminal domains. Additionally, we detected interacting phenotypes when RecA and YabT, fused to AD and BD-Gal4 domains, respectively, were coexpressed in the yeast cell (Fig. 5D). This interaction also requires the integrity of RecA, since it was abolished by deletion of either N- or C-terminal domains of RecA. These observations support the interaction between YabT and RecA *in vivo*. Interestingly, point mutants of RecA at serine 2 (RecA S2A and RecA S2D) were not able to interact with YabT, suggesting that specific recognition of RecA serine 2 might be important for this interaction. To rule out the possibility that the residue serine 2 is only essential for interaction of RecA with YabT, but not necessarily phosphorylated itself, we performed a mass spectrometry analysis of C-terminally 6xHis-tagged wild type RecA phosphorylated *in vitro* by YabT. The result confirmed unambiguously that RecA is indeed phosphorylated on serine 2 (Fig. S3), the same residue that was found to be phosphorylated *in vivo* (Soufi *et al*., 2010).

### Loss of RecA phosphorylation leads to the same phenotype as the loss of YabT

If RecA is the substrate of YabT *in vivo*, the loss of phosphorylation at its serine 2 should be expected to have similar consequences as the loss of the kinase YabT. To test this hypothesis in living *B. subtilis* cells, we constructed two point mutants at the *recA* locus: the non-phosphorylatable *recA* S2A, and the mutant *recA* S2D, which with its negatively charged side-chain mimics phosphorylation. *recA* S2D would thus be expected to restore or compensate any loss of function observed in *recA* S2A or Δ*yabT*. To make sure that these point mutations did not interfere with the overall RecA protein expression or stability, we checked the *in vivo* levels of RecA in all strains used for our physiological assays (Fig. 5E). RecA levels were probed in crude extracts using an anti-RecA antibody. Δ*recA* strain was used as a negative control, and no RecA signal was detected. RecA levels were identical in the wild type, Δ*yabT*, *recA* S2A and *recA* S2D strain. Since the mutant proteins RecA S2A and RecA S2D also retained their ability to self-interact in the two hybrid assay (Fig. 5D), we were satisfied that these point-mutant proteins are properly expressed and folded.

Our first aim was to verify whether modifications of serine 2 would have any phenotype in standard assays related to generally known roles of RecA such as DNA recombination and DNA lesion repair. Wild type, *recA* S2A and *recA* S2D mutant strains performed homologous recombination with various lengths of homologous DNA substrates with equal efficiency (Fig. S4A). The ability of *recA* S2A and *recA* S2D to cope with DNA damage induced in a standard assay with mitomycin C added during exponential growth was equal to the wild type (Fig. S4B). Next we examined the ability of *recA* S2A and *recA* S2D to induce the SOS response via LexA cleavage (Butala *et al*., 2009). Again, *recA* S2A and *recA* S2D behaved exactly as the wild type (Fig. S4C). Δ*yabT* also had no effect on SOS induction. Accordingly, wild type RecA, RecA S2A and RecA S2D had a comparable capacity to promote LexA cleavage *in vitro* (Fig. S4D). At this point we concluded that the functional significance of RecA phosphorylation should be explored outside vegetative growth. We thus turned to sporulation, and the conditions in which *yabT* expression reaches its maximum.

In all our sporulation-related assays, the phenotype of the non-phosphorylatable *recA* S2A matched that of Δ*yabT*. With respect to reduced speed of spore formation, both strains produced only 30–35% of heat resistant spores 7 h after the initiation of spore development (Fig. 4A). They also had a similar drop of spore survival when exposed to mitomycin at the onset of sporulation (Fig. 4B). By contrast, the phospho-mimetic *recA* S2D was very similar to wild-type cells in terms of sporulation kinetics (80% of heat-resistant spores after 7 h) and spore survival after mitomycin treatment. To confirm that *ΔyabT* phenotype is indeed related to RecA phosphorylation, and not to some unrelated YabT function, we introduced the *recAS2D* mutation into *ΔyabT* cells. This restored their sporulation kinetics and spores resistance to mitomycin to the levels of the wild type cells or the single *recAS2D* mutant respectively. This behaviour is consistent with the idea that YabT acts during spore development via phosphorylation of RecA at serine 2. Phosphorylation of RecA plays no role in its functions during exponential growth, so what is different for RecA during sporulation?

### RecA forms a transient, mobile focus associated with the chromosome during spore development

In order to better understand the role of RecA during spore development, we decided to follow its localization using RecA fused to GFP. Several such fusions optimized for *B. subtilis* have been reported previously (Kidane and Graumann, 2005; Meile *et al*., 2006; Simmons *et al*., 2007), and none of them are entirely free of artefacts. The workable strains expressing a GFP-tagged RecA were carrying either a fusion at the natural *recA* locus (Simmons *et al*., 2007), or ectopic GFP fusions in the presence of a wild type *recA* allele (Kidane and Graumann, 2005; Meile *et al*., 2006) (see Table S1). Of note, cells expressing these fusions as the sole source of RecA remained deficient for survival under severe DNA damaging conditions during vegetative growth, indicating that they were not fully functional (Meile *et al*., 2006; Simmons *et al*., 2007). However, both fusions were reported to localize in a replication dependant manner during vegetative growth (Meile *et al*., 2006; Simmons *et al*., 2007). Since chromosomal replication ceases around 1 h after the onset of sporulation, we first asked whether RecA retains its ability to form foci during this developmental process.

**Fig. 6:**
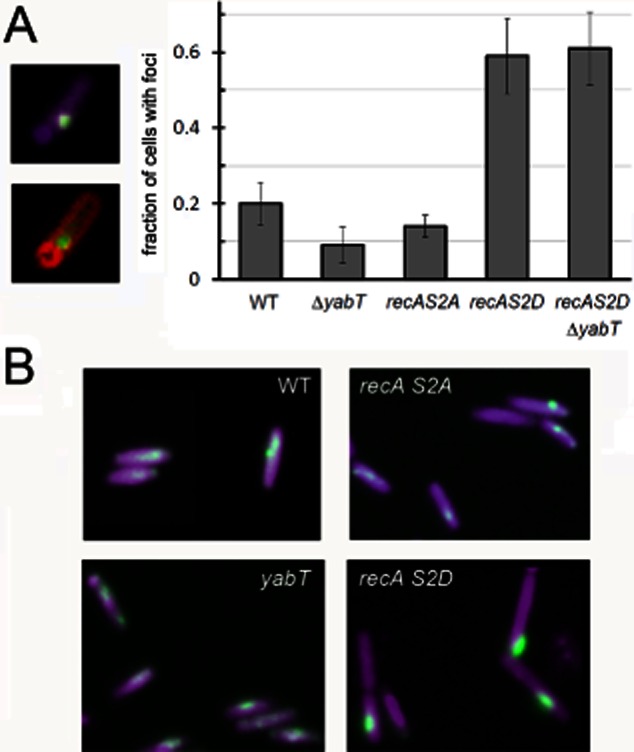
RecA foci observed during spore development. A. GFP-RecA foci visualized by fluorescent microscopy at T3. The overlay pictures of the same cell represent membrane-staining (FM4-64) in red, nucleoid (DAPI) in violet and GFP-RecA is green. The GFP-RecA fusion was induced at T0. The proportion of cells containing GFP-RecA foci at T3 is shown in wild type and different mutant stains: Δ*yabT*, *recA* S2A, *recA* S2D and *recA* S2D Δ*yabT*. Detection of foci for counting was done with the help of the fluorescent spot detector software (FluorSpotRecognition). Error bars represent standard deviation from 3 independent experiments. B. Externally induced DNA damage transforms RecA foci into filaments/threads. DNA damage was induced at T1 with 40 ng ml^−1^ of mitomycin. Cells were observed with FITC/DAPI-staining overlap at T3. Strain names are indicated in each panel.

We tested several available chimerical combinations of RecA and GFP for detecting RecA localization during spore development, while seeking to maintain wild type level of cellular resistance to severe DNA damage. The overview of these results is given in Table S1. We first tested a RecA C-terminally fused to an engineered monomeric GFP-variant (RecA-GFP) reported to be proficient for DNA repair at low doses of exogenous DNA-damaging agents during vegetative growth and which exhibits DNA-damaged induced foci (Simmons *et al*., 2007). We did not observe any localization pattern in this strain during sporulation (Table S1). To circumvent the problem of functionality, we looked at the *recA-GFP* fusions expressed ectopically in a strain harbouring the wild type genomic copy of *recA* (Kidane and Graumann, 2005; Meile *et al*., 2006; Simmons *et al*., 2007). In this genetic context, the genomic copy of *recA* ensures proper vegetative growth and all cellular functions of RecA. The *gfp*-tagged copy of *recA* is induced only at the onset of sporulation in order to infer the localization of the non-tagged RecA, via formation of a mixed nucleoprotein complex RecA/GFP-RecA. We ruled out the use of the monomeric GFP and C-terminal fusions, since upon induction at the onset of sporulation they produced a fluorescent inclusion body at the cell pole, sometimes in addition to a focus (Fig. S5A). Hence, the only construct that remained fully functional and provided meaningful localization was the N-terminal fusion expressed ectopically from the *amyE* locus in the presence of the wild type *recA* allele (*recA*+ *amyE*::Pxyl::*gfp-recA*) (Meile *et al*., 2006; Fig. S5B, Table S1). In this strain the induced GFP-RecA had intracellular levels similar to the wild type RecA expressed from the endogenous *recA* allele and the strain had wild type levels of survival under severe DNA damage (Fig. S6). At stage T3 of sporulation, in the absence of exogenous DNA damage, we observed about 20% of the cells exhibiting a single RecA focus (Fig. 6A). Surprisingly, in some cells the RecA foci were moving rapidly, scampering around the mother cell in a scanning-like movement (Movie S1). The RecA focus remained in the mother cell and appeared transiently associated with the nucleoid (Movie S2). Such a dynamics is reminiscent to that of the checkpoint protein DisA that monitors chromosome integrity at the early stage of sporulation (Bejerano-Sagie *et al*., 2006). Upon treatment with mitomycin, in most cells, these moving foci were transformed into fluorescent thread-like structures that retained some dynamism across the nucleoids (Fig. 6B, Movie S3). RecA threads have been also observed in response to DNA damage during vegetative growth (Kidane and Graumann, 2005; Simmons *et al*., 2007). Together these observations indicate that the RecA dynamic foci are biologically relevant and that the GFP-RecA can form a RecA nucleofilament in response to exogenous DNA damage. This nucleofilament is actually driven by the non-tagged RecA expressed from the locus. Thus it is consistent to propose that both thread-like structures and dynamic foci contain mixed RecA/GFP-RecA complexes.

**Fig. 7:**
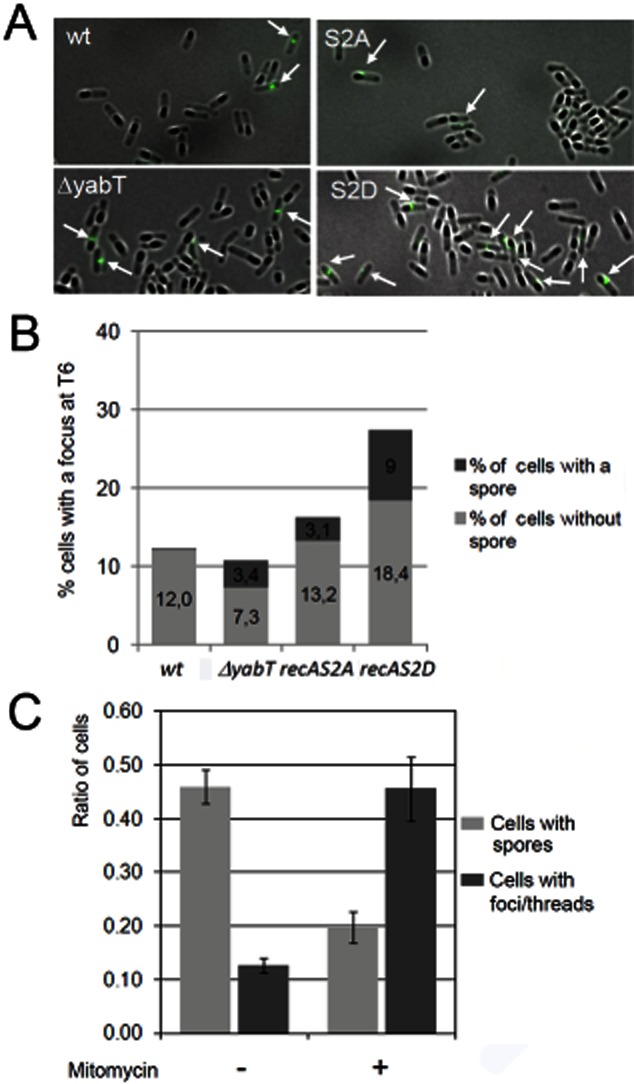
RecA foci persisting at T6 are incompatible with sporulation in wild type cells. A. A sample of sporulating wild type cells observed at T6 with FITC/brightfield overlap. Green spots in FITC/brightfield overlap represent RecA foci and black spots are mature spores. Strains are indicated in each panel. White arrows highlight the cells containing RecA foci. B. Coexistence of RecA foci and spores at T6 in different strains. Over 500 cells were counted for each strain. Total bar height represents the fraction of cells containing a RecA focus. Light-grey bar represents the absence of a spore and dark grey bare the presence of a spore in cells containing RecA foci. C. Over 500 wild type cells were examined to count the ratio of cells with spores and cells with RecA foci/threads at T6. To the left is the ratio in the untreated wild type cells, and to the right the ratio in wild type cells treated with mitomycin.

In the absence of DNA damage, by the time the spore development completes around T6, RecA foci disappear in most cells. However, a closer examination of few *B. subtilis* cells with foci remaining at T6 revealed one interesting feature. None of the mother cells that have successfully produced a spore contained the RecA focus. Foci that persist at T6 are found exclusively in the cells that have not produced mature spores (Fig. 7A). This suggested that persistent RecA foci and accomplished spore development are mutually exclusive. Persistent foci have been previously described in exponentially growing cells as associated to replication forks stalled at irreparable lesions (Kidane and Graumann, 2005; Simmons *et al*., 2007). It is therefore tempting to speculate that RecA foci persisting at T6 could also correspond to irreparable lesions. If this is true, then exogenous DNA damage should lead to more persistent foci at T6 and less spores. To test this hypothesis, we examined the T6 samples of wild type cells that were treated with mitomycin at T1 (Fig. 7C). In the mitomycin-treated culture, the ratio of mother cells with RecA foci to cells with mature spores at T6 inverted sharply. While the spores and foci remained mutually exclusive, the majority of cells now contained RecA assemblies and the fraction of mature spores diminished strongly (Fig. 7C). This finding suggests a link between RecA assemblies persistent at T6, irreparable DNA lesions and failure to complete a mature spore.

### The effect of phosphorylation on RecA foci during spore development

Next we explored the possibility that RecA phosphorylation and RecA focus formation might be correlated. The construct *amyE*::Pxyl::*gfp-recA* has therefore been transferred in the Δ*yabT* strain. As for the non-phosphorylatable S2A and phosphomimetic S2D mutations, they were inserted in the *gfp-recA* fusion and coexpressed together with their cognate *recA* S2A and *recA* S2D genomic copies. These strains were then used to follow RecA localization profile during sporulation. As mentioned above, RecA mutations S2A and S2D did not interfere with the classical RecA functions in DNA recombination, repair, and SOS induction, during exponential growth. At the onset of sporulation, mobile GFP-RecA foci appeared in a subset of cells in all strains. A statistical analysis of the foci velocity revealed that it is roughly similar in all strains (Fig. S7). As mentioned before, at T3 about 20% of wild type cells contained RecA foci (Fig. 6A). The number of foci was slightly reduced in Δ*yabT* and *recA* S2A strains, and it markedly increased in *recA* S2D strain, with about 60% of all examined cells presenting a RecA focus. The combined mutant strain *recA* S2D Δ*yabT* also exhibited about 60% cells with foci. This is again consistent with phosphorylated RecA being downstream of YabT in the regulatory pathway. Therefore, we concluded that in the absence of exogenous DNA damage, RecA phosphorylation increases the RecA propensity to form foci during sporulation. Since Δ*yabT* and *recA* S2A strains exhibited a moderate decrease in number of foci, and *recA* S2D a more dramatic increase with respect to wild type, we speculate that the stoichiometry of RecA phosphorylation in the wild type is not very high. Similar to what was observed for the wild type GFP-RecA, the morphology of foci shifted from clearly defined spots to thread-like structures, upon inducing exogenous DNA damage in Δ*yabT* and *recA* S2A strains (Fig. 6B). The only strain exempt from this transformation was the *recA* S2D, which maintained bright individual foci in most cells.

The loss of phosphorylation in Δ*yabT* and *recA* S2A strains lead to fewer foci at T3 (Fig. 6A) and a decreased survival under external DNA damage (Fig. 4B). Phosphorylation also affected the incompatibility between persistent RecA foci and mature spores at T6. This incompatibility was relaxed in the Δ*yabT*, *recA*S2A and *recA* S2D mutant strains, in which a new sub-population of cells appeared, containing both persisting foci and fully developed spores (Fig. 7B). This might suggest that wild type levels of RecA phosphorylation are required to ensure the exclusivity between mature spores and persistent foci. If this equilibrium is disrupted, either by loss of RecA phosphorylation (Δ*yabT* or *recA* S2A), or excessive phosphorylation (*recA* S2D), spore-bearing cells with foci appear. Thus, one might speculate that RecA phosphorylation could contribute to a checkpoint that prevents the completion of spore development in mother cells with severely damaged chromosomes. In this context, phosphorylation of RecA that increases the propensity of foci formation could be seen as a contributory mechanism. More work will clearly be required to address the role of RecA in spore formation and the underlying role of phosphorylation in this mechanism.

## Conclusions

In *B. subtilis*, the serine/threonine kinase YabT, devoid of extracellular sensing domain, is expressed specifically during spore development and exhibits septal localization. Our *in vitro* data show that YabT auto-activates upon binding DNA, and specifically phosphorylates RecA at the residue serine 2. RecA and YabT interact in a two-hybrid assay, and loss of either the kinase or the RecA phosphorylated residue S2 lead to the same phenotype *in vivo*: retarded sporulation and drop in DNA damage resistance during spore development. Given that YabT is localized at the septum, it might encounter RecA associated to the DNA, while the chromosome is transported through the septum. However, the possibility that YabT also phosphorylates cytosolic RecA cannot be excluded. Phosphorylation of RecA seems to facilitate the formation of RecA foci in *B. subtilis* cells. The molecular mechanism of RecA recruitment to the nucleoid of the mother cell and the dynamics of RecA foci are presently not understood. However, if the RecA focus remains until T6 it coincides with absence of spore. Such persistent focus might correspond to an irreparable lesion site, and RecA phosphorylation may play a role in delaying or aborting sporulation in response to DNA damage. A similar function has been described for the c-di-AMP cyclase checkpoint protein DisA, which also forms a cell-cycle phase-dependent focus at the onset of sporulation, sensing for the presence of stalled DNA structures (Bejerano-Sagie *et al*., 2006; Witte *et al*., 2008; Oppenheimer-Shaanan *et al*., 2011). Yet, this role of RecA regulated by phosphorylation during sporulation is novel, and we intend to devote our attention to dissecting this mechanism in subsequent studies.

The absence of a YabT orthologue and the RecA residue S2 in *E. coli* and other non-sporulating bacteria (while both YabT and S2 of RecA are conserved in the majority of *Bacilli*) corroborates the idea that this mechanism evolved for the specific task of ensuring chromosome integrity during spore development in this genus. Parallels that can be drawn between YabT and the eukaryal kinases C-Abl and Mec1 support the notion that the mechanisms governing bacterial development share similarities with their counterparts in *Eukarya*, where protein phosphorylation is commonplace. Thus RecA shares not only the functional similarity with its eukaryal homologue Rad51, but also similarity of regulation via post-translational modifications.

## Experimental procedures

### Strain construction and growth conditions

Cells were routinely grown in LB medium containing, when needed, spectinomycin 60 μg ml^−1^, phleomycin 2 μg ml^−1^, neomycin 5 μg ml^−1^, chloramphenicol 7 μg ml^−1^, erythromycin 0.5 μg ml^−1^ or ampicillin 100 μg ml^−1^. *E. coli* TG1 was used for plasmids construction. *B. subtilis* strains used in the study are constructed at the basis of the TF8a (Westers *et al*., 2003) derivative BS33 containing a neomycin resistance gene under the control of the Lambda Pr promoter (λPr-*neo*) (Itaya, 1999) (Table S2). The PCR primers used in this study are listed in Table S3. To construct the knock-out *yabT* mutant, the DNA fragments flanking *yabT* gene were amplified with the primer pairs mra5 + mra7F and mra6 + mra7R and fused to the extremities of the phleomycin-resistance gene using PCR with primers mra5 and mra6. The resulting fragment was used to transform BS33 cells, and transformants were selected for phleomycin-resistance. The *yabT* deletion marked with the spectinomycin-resistance marker was constructed in the same way. Primers *pyabT*Apa and *pyabT*Sal were used to amplify the *yabT* gene, which was cloned between ApaI and SalI sites in pSG1729 (Lewis and Marston, 1999), to obtain N-terminal GFP-*yabT* fusion at the *amyE* locus. The GFP fusion with the YabT mutant lacking the putative transmembrane domain was constructed similarly using primers *pyabT*Apa and *pyabTΔTM*. The fusion of *yabT* gene with SPA tag was obtained by amplification of the C-terminal part of *yabT* with primers *pyabT*Bam and *pyabT*Nco and the product was integrated between BglII and NcoI sites in pMUTIN-SPA (Lecointe *et al*., 2007). The *recAS2A* and *recAS2D* mutants were constructed at locus by an improved mutation delivery approach (Fabret *et al*., 2002; Tanaka *et al*., 2012) as follows. The primer pairs mra1 + mra3R and mra2 + mra3F were used to PCR-amplify the partially overlapping DNA fragments containing the parts of *recAS2A* gene and the flanking regions. Mra1 + mra4R and mra2 + mra4F were used for *recAS2D*. The insertion cassette encoding the Lambda CI repressor and the phleomycin-resistance gene was flanked by the mutagenized *recA* fragments in direct orientation by PCR with primers mra1 and mra2 and used to transform BS33 cells with selection on phleomycin. The obtained clones were controlled for a loss of neomycin-resistance due to CI-mediated repression of the Pr promoter. Finally, the cells in which the cassette was lost due to recombination between the direct repeats were isolated by counter-selection for neomycin-resistance. For SOS assays, the strains containing *recA* wild type and mutants alleles were transformed with DNA bearing the promoter fusion *P_dinR_::lacZ* (Duigou *et al*., 2004). The N-terminal fusions of RecA, RecAS2A, RecAS2D proteins with GFP were constructed at the chromosomal *amyE* locus similarly to GFP-*yabT* (see above) using the universal primer in pairs with p*recA*wt, p*recA*S2A and p*recA*S2D respectively. For yeast two hybrid analysis, the entire *recA*, *recAS2A* and *recAS2D* genes were amplified using the reverse primer mra9 paired with the forward primers p2HRwt, p2HRS2A and p2HRS2D respectively. Products were cloned in pGAD-c2 and pGBDU-c2 plasmids between BamHI and PstI sites. The *yabT* gene was cloned in similar manner using the primers pair p2HyabT-F + p2HyabT-R and the restriction enzymes SmaI and SalI.

### Synthesis and purification of affinity-tagged proteins

For protein purification, RecA_wt forward, RecA_S2A forward or RecA_S2D forward primers were used in combination with the RecA reverse primer to obtain respective *recA* versions. We prepared the N- and C-terminal fusions of 6x-His tagged RecA. YabT-specific primers were used for amplifying different versions of the kinase gene: wild type, truncated and K55D, and also *gfp-yabT*. LexA primers were used to amplify *lexA* gene (Table S3). PCR was performed with genomic DNA of *B. subtilis* 168, except *gfp-yabT* which was amplified from BMR154 strain. All PCR fragments were cloned in pQE30 (Qiagen) to obtain the 6xHis-tag fusion proteins, except the C-terminal His_6_ fusion of *recA* which was inserted in pQE-60. For the protein kinases PrkC and YabT, only soluble cytosolic domains were cloned. Strep-tagged version of *yabT* was prepared by cloning its gene in the pQE-30 vector with His_6_ tag replaced by the strep-tag (Jers *et al*., 2010). For all proteins synthesis and purification were performed using the standard protocol described previously (Mijakovic *et al*., 2003). Briefly, expression was induced at OD_600_ 0.6, cells were harvested 3 h later and sonicated. From crude extracts, 6xHis-tagged proteins were purified using Ni-NTA affinity chromatography (Qiagen), and desalted using PD-10 columns (GE Healthcare).

### Protein phosphorylation assay

For *in vitro* phosphorylation assays, reactions were started by adding 50 μM ATP containing 20 μCi mmol^−1^ [γ-^32^P]-ATP, and radioactive phosphorylated proteins were revealed by autoradiography using a phosphoimager from FUJI, as described previously (Mijakovic *et al*., 2003). Protein concentrations and incubation times in all assays are specified in figure legends. Three independent experiments were performed for each assay, and a representative sample is shown. Signal quantification was performed by Fiji (http://fiji.sc/), and mean values from 3 independent experiments are shown.

### Electrophoretic mobility shift assays

The electrophoretic mobility shift assays were performed in the reactions containing 25 mM Tris-HCl, pH 7.5, 50 mM NaCl, 5% glycerol, 1 mM DTT, 10 mM MgCl_2_, 50 μg ml^−1^ BSA, 1 mM ATP, 140 nucleotide-long ssDNA or 210 base pair dsDNA and protein (DNA and protein concentrations are indicated in the figure legends). 140 ssDNA was a random sequence primer, and 210 bp dsDNA was the PCR product obtained from *B. subtilis* genomic DNA by using primers NCterF and NCterR from Mijakovic *et al*. (2003). Reactions were incubated at 37°C for 30 min, and analysed by gel electrophoresis in 1.0–1.5% agarose gels. Migration was performed for 2 h, at 2 V cm^−1^ and 4°C, and the gel was stained with Ethidium Bromide. Three independent experiments were performed, and a representative sample is shown.

### Fluorescence microscopy: detection of GFP-YabT and GFP-RecA

Sporulation was initiated as previously described (Nicholson and Setlow, 1990). Briefly, an aliquot of 100 μl of exponentially growing cells (LB at 37°C) was spread onto DSM plates (Schaeffer *et al*., 1965), and grown over-night at 30°C. Colonies were resuspended in liquid DSM supplemented with appropriate antibiotic and OD_600_ adjusted to ∼ 0.1. Cells were then grown at 37°C. 0.5% xylose was added to induce the expression of *gfp-yabT* or *gfp-recA* (either during exponential growth or at T0). For GFP-YabT samples were taken at OD_600_ 0.6 for exponential phase and at T3 (after a 3 h induction) for sporulation, and examined by fluorescence microscopy. Fluorescence was quantified using ImageJ (Fiji). A total of 303 cells were examined for BMR154 (GFP-YabT) during sporulation, 200 cells for BMR154 (GFP-YabTΔTM) during sporulation and 218 cells for BMR154 (GFP-YabT) during exponential phase. For GFP-RecA, samples were taken at T1, T2, T3, T5 and T6. Cells were rinsed in MMS and mounted on 1.2% agarose pads. When required, cells were stained with FM4–64 (Molecular Probes) to visualize the cell membrane or DAPI to visualize the nucleoid. Fluorescence microscopy was performed on a Leica DMR2A (100 UplanAPO objective with an aperture of 1.35 and a CoolSnap HQ camera (Roper Scientific) and on a Nikon Eclipse Ti (100 FluoPlan objective with an aperture of 1.30 and a ORCA R2 camera (Hamamatsu)). System control and image processing were performed using MetaMorph software. Three independent experiments were performed for each strain, observing a minimum of 500 cells.

### Western blotting

The crude cell extracts were prepared as described (Lecointe *et al*., 2007). Bradford assay was used to determine total protein concentration in each extract. An equal total amount of protein for every strain was loaded and separated by SDS-PAGE (12% polyacrylamide). The RecA proteins were visualized using the primary rabbit serum against the *E. coli* RecA (S. Marsin, CEA, France; working dilution 1:5000) and the secondary goat peroxidise-coupled anti-rabbit IgG (Sigma; dilution 1:10 000). The SPA-tagged YabT was visualized using the primary mouse ANTI-FLAG M2 monoclonal antibodies (Sigma; dilution 1:5000) and the secondary goat peroxidise-coupled anti-mouse IgG antibodies (Sigma; dilution 1:20 000). Three independent experiments were performed, and a representative sample is shown.

### Sporulation assay

Cells grown over-night on the solid DSM medium (Schaeffer *et al*., 1965) were inoculated in the preheated liquid DSM at OD_600_ 0.1 and incubated at 37°C until OD_600_ 1.5. From this point (taken as T0) samples were grown for 20 h and plated in dilutions on LB. Half of the material was heated at 80°C (10 min) before plating. Colonies were counted after 36 h of incubation at 37°C, and the percentage of spores was calculated as the ratio of colonies forming units in heated and unheated samples. The growth curves of outgrowing cultures were the same for all strains. Five independent experiments were performed. For mitomycin treatment, spores were grown as described previously (Leighton and Doi, 1971). At T1, the culture was split in two: 20 ng ml^−1^ mitomycin C (final concentration) was added to a half of the culture and the other half was used as control. At T7, both treated and untreated cultures were analysed for spore formation as described above. Five independent experiments were performed.

### Recombination assay

To test DNA recombination by single crossing-over, the competent *B. subtilis* wild type, the non-phosphorylatable *recAS2A* and the phosphomimetic *recAS2D* mutant cells were transformed by the same amounts of the integrative plasmid pMUTIN2 containing different fragments of the *B. subtilis* chromosome. To test DNA recombination by double crossing-over they were transformed with *B. subtilis* genomic DNA containing inserted pMUTIN2 plasmid. The efficiency of transformation was determined as the ratio of the erythromycin resistant colonies to the number of viable cells in competent cultures. Three independent experiments were performed with each strain.

### Induction of the SOS response

The cells containing wild type *recA*, *recA* S2A or *recA* S2D, together with a transcriptional fusion *P_dinR_::lacZ* were grown to early exponential phase (OD_600_ 0.03). SOS response was induced by 40 ng ml^−1^ of mitomycin. Cells were harvested at different times after induction, β-galactosidase activity was recorded and plotted against time after induction. Five independent experiments were performed.

### LexA cleavage assay

LexA cleavage assay was performed as described previously (Harmon *et al*., 1996). Purified proteins were incubated in a buffer containing 25 mM Tris-Cl, pH 7.5, 50 mM NaCl, 5% glycerol, 1 mM DTT, 10 mM MgCl_2_, 5 mM ATP, 2 mM dATP, 20 μg ml^−1^ BSA at 37°C. 1.5 μM RecA (wild type or mutant) were pre-incubated with 40 ng M13 phage ssDNA for 10 min before the reactions were initiated by adding 4.5 μM LexA into the reaction mixture. LexA cleavage was visualised by SDS-PAGE (12% polyacrylamide) and staining by Coomassie blue.

### Yeast two hybrid

The yeast two hybrid phenotypic assay for YabT-RecA interaction was performed as described previously (Noirot-Gros *et al*., 2002). Briefly, the following gene fusions were produced: *recA* wild type and mutant versions with the activating and the DNA-binding domain of Gal4, and *yabT* with the DNA-binding domain of Gal4. Resulting plasmids were inserted in yeast haploid cells and interacting phenotypes were screened for ability to grow on the selective medium (-LUH). Three independent experiments were performed.

### Nano LC-MS/MS analysis of purified phosphorylated RecA

*In vitro* phosphorylation reaction of RecA in the presence of YabT was set up as described above, with the only difference of using non-radioactive ATP. C-terminally 6xHis tagged wild type RecA was used. After SDS-PAGE, in-gel digestion was performed for 6 h at 37°C with 100 ng of modified trypsin (Promega) dissolved in 50 mM NH_4_CO_3_. The peptides were extracted successively with 2% trifluoroacetic acid (TFA) and 50% acetonitrile (ACN) and then with ACN. Peptide extracts were dried in a vacuum centrifuge and suspended in 30 μl of 0.05% TFA, 0.05% HCOOH, and 2% ACN. HPLC was performed on a NanoLC-Ultra system (Eksigent). Eluted peptides were analysed on-line with a QExactive mass spectrometer (Thermo Electron) using a nanoelectrospray interface. Peptide ions were analysed using Xcalibur 2.1 with the following data-dependent acquisition steps: (i) full MS scan [mass-to-charge ratio (m/z) 400 to 1400, resolution 70 000] and (ii) MS/MS (normalized collision energy = 25%, resolution 17 500). Step 2 was repeated for the 8 major ions detected in step 1. Dynamic exclusion was set to 40 s. A database search was performed with XTandem (version 2011.12.01.1) (http://www.thegpm.org/TANDEM/) and confirmed with MaxQuant (http://www.maxquant.org/). Cys carboxyamidomethylation and Met oxidation were set to static and possible modifications respectively. Precursor mass was 10 ppm and fragment mass tolerance was 0.02 Th. Identified phosphopeptides were filtered and grouped using XTandem Pipeline (http://pappso.inra.fr/bioinfo/xtandempipeline/) and manually validated.
