# Supplementary material for: Bacillus subtilis serine/threonine protein kinase YabT is involved in spore development via phosphorylation of a bacterial recombinase
Source: Mol Microbiol. 2013 May 2;88(5):921–35. doi: 10.1111/mmi.12233 (PMC3708118; doi:10.1111/mmi.12233)
Supplement: Supplementary file 1 [file mmi0088-0921-SD1.zip › mmi_12233_Supporting Information.pdf]

SUPPORTING INFORMATION

Bacillus subtilis serine/threonine protein kinase YabT is involved in spore development via phosphorylation of a bacterial recombinase

Vladimir Bidnenko[1,¶], Lei Shi[1,¶], Ahasanul Kobir[1], Magali Ventroux[1], Nathalie Pigeonneau[1], Céline Henry[1], Alain Trubuil[3], Marie-Françoise Noirot-Gros[1,*] and Ivan Mijakovic[1,2*]

[1] INRA, UMR1319 Micalis, Jouy-en-Josas, F-78350, France

[2]AgroParisTech, UMR1319 Micalis, Jouy-en-Josas, F-78350, France

[3] Unité de recherche Mathématiques et Informatique Appliquées, INRA, Jouy-en-Josas, FR-78350, France

[¶]These authors contributed equally to this study.

* To whom correspondence should be addressed.

Prof. Ivan Mijakovic, PhD: Tel: +33 1 30 81 45 40, Fax: +33 1 30 81 54 57, E-mail: Ivan.Mijakovic@grignon.inra.fr

Marie-Françoise Noirot-Gros, PhD: Tel: +33 1 34 65 25 19, Fax: +33 1 34 65 72 44, E-mail: marie-francoise.gros@jouy.inra.fr

SUPPORTING EXPERIMENTAL PROCEDURES

Particle tracking

Image processing was performed using the Image Processing Toolbox of MATLAB (The Mathworks). To identify fluorescent foci, time lapse images were processed as follows (the source code is available upon request): images were firstly denoised with ND-SAFIR (Boulanger *et al.*, 2010). The denoised images were used to detect regional extrema. A combination of morphological operations was performed on the extrema images to identify foci candidates. Background fluorescence is subtracted from the total fluorescence of the foci. To track fluorescent foci, each cell is checked for the recognition of a focus at each time step. Once the foci trajectories were determined, several analyses were performed: mean foci position during the time period, mean and standard deviation of displacement between two consecutive time steps, maximal euclidean distance between two positions, cell main axis length, ratio of maximal distance and axis length, mean foci speed during time period, mean foci fluorescent intensity during time period.

SUPPORTING FIGURES

Figure S1

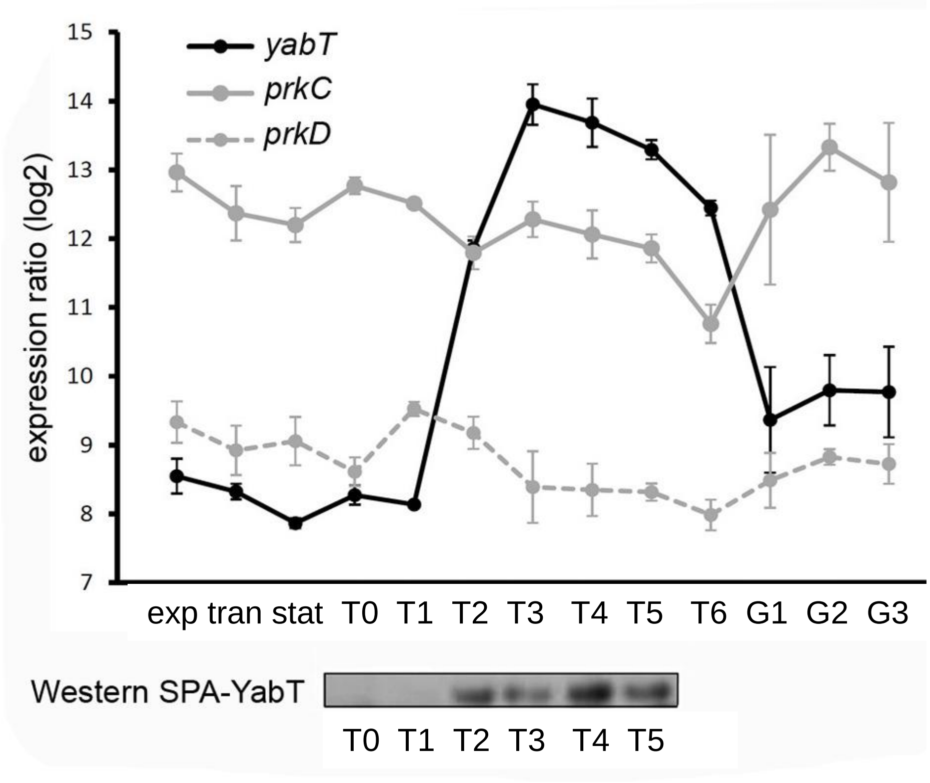

Figure S1. YabT is overexpressed during sporulation. Expression profile of *yabT*. *B. subtilis* transcriptome data (Nicolas *et al.*, 2012) presented as Log_2 normalized expression ratios are shown for *yabT*, *prkC* and *prkD* during vegetative growth, spore development (T0-T6) and germination (G1-G3). SPA-tagged YabT was expressed from the native *yabT* promoter for the purpose of detection by anti-SPA antibody in Western blot. The protein was detected between T2-T5.

Figure S2

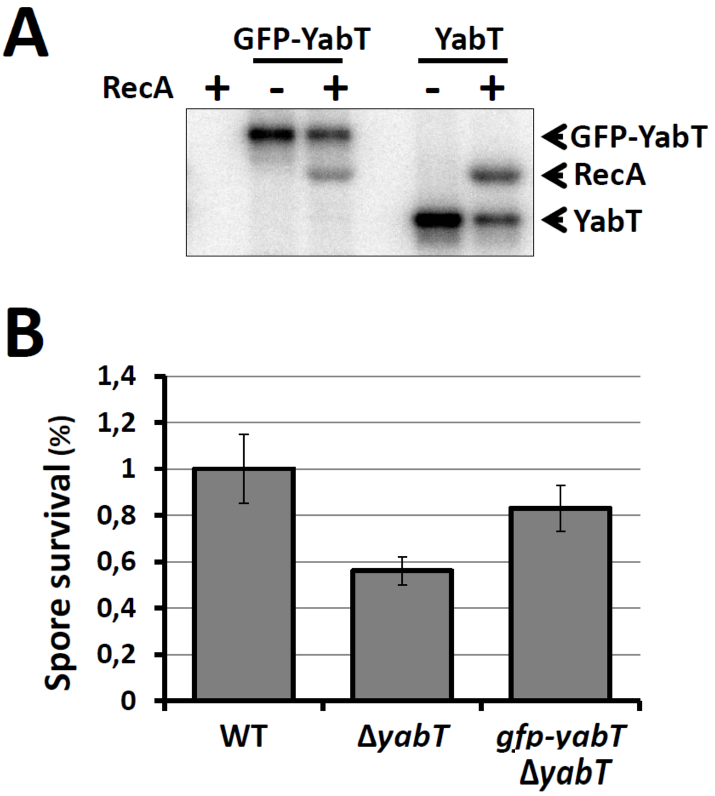

Figure S2. Functional assays with the GFP-YabT fusion. (A) (D) *In vitro* phosphorylation assay showing the autophosphorylation of GFP-YabT and phosphorylation of the substrate RecA by GFP-YabT. RecA incubated without the kinase is shown as control. Reactions contained 1 µM RecA and 0.25 µM GFP-YabT and YabT and were incubated for 2 h prior to separation by SDS-PAGE. Bands corresponding to autophosphorylated YabT, GFP-YabT and phosphorylated RecA are indicated by arrows. (B) Spore survival after mitomycin treatment (20 ng ml^{-1}) applied at time-point T1 to the sporulating cultures of *B. subtilis* wild type, Δ*yabT* (BMR 25), and Δ*yabT* P_{xyl}*::gfp-yabT* (BMR154). Spore counts are expressed as number of spores in treated culture/number of spores in untreated culture for each strain, normalized with respect to the wild type. Error bars represent standard deviation from 5 biological replicates.

Figure S3

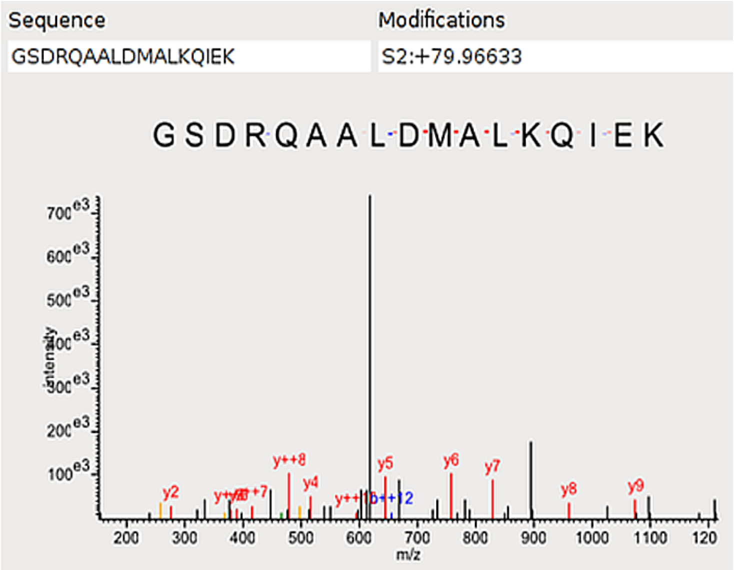

Figure S3. Mass spectrometry detection of the phosphorylation site on RecA. *In vitro* phosphorylation reaction of RecA was carried out in the presence of YabT as described in Experimental Procedures, only with non-radioactive ATP. C-terminally 6xHis tagged wild type RecA was used, which has an extra glycine residue inserted between the N-terminal methionine and serine 2 (due to restriction site constraints of the vector pQE-60). Identification of the phosphorylation site was performed on the Q Exactive instrument as described in Experimental Procedures. The result shows an 80 Da molecular weight increment (corresponding to phosphate) at the position of serine 2.

Figure S4

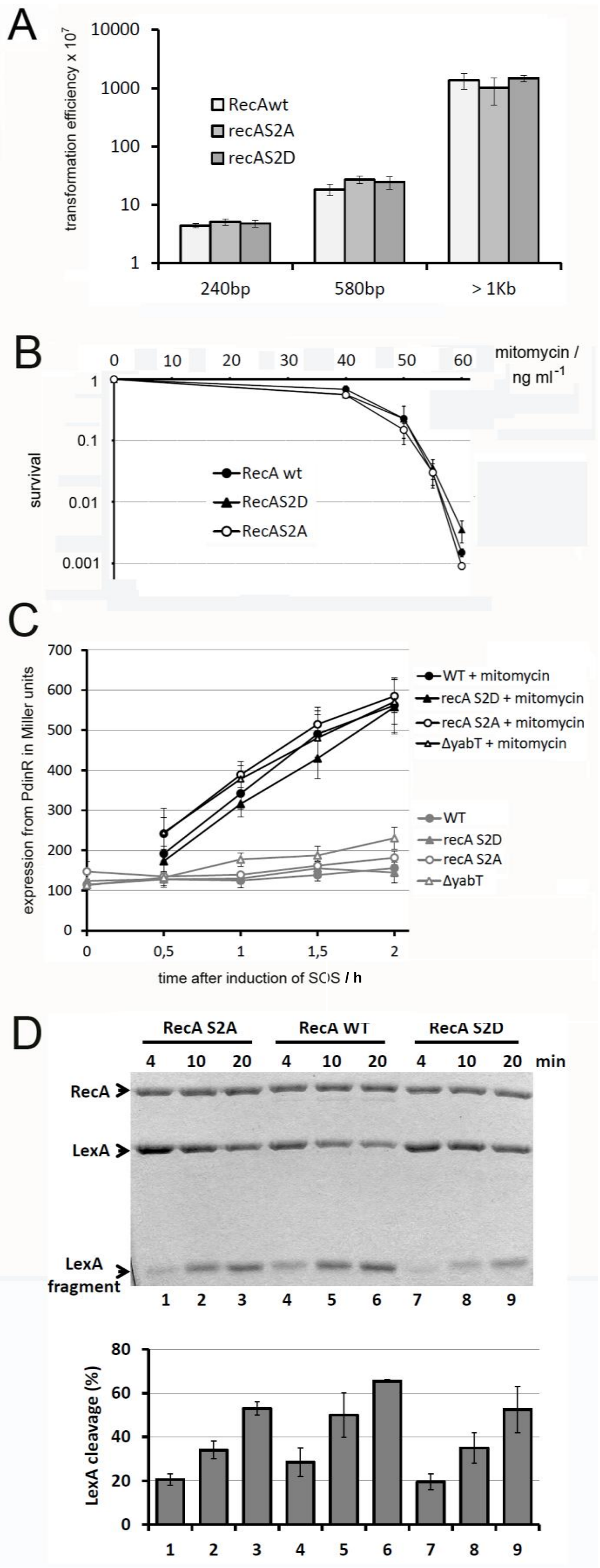

A
transformation efficiency x 10^7
10000
1000
100
10
1
RecAwt
recAS2A
recAS2D
240bp
580bp
> 1Kb
B
0
10
20
30
40
50
60
mitomycin / ng ml^{-1}
survival
1
0.1
0.01
0.001
RecA wt
RecAS2D
RecAS2A
C
expression from PdinR in Miller units
700
600
500
400
300
200
100
0
WT + mitomycin
recA S2D + mitomycin
recA S2A + mitomycin
ΔyabT + mitomycin
WT
recA S2D
recA S2A
ΔyabT
0
0,5
1
1,5
2
time after induction of SOS / h
D
RecA S2A
RecA WT
RecA S2D
4 10 20 4 10 20 4 10 20 min
RecA
LexA
LexA fragment
1 2 3 4 5 6 7 8 9
LexA cleavage (%)
80
60
40
20
0
1 2 3 4 5 6 7 8 9

Figure S4. RecA phosphorylation does not influence DNA damage repair or homologous recombination in vegetative cells. (A) To test DNA recombination by single crossing-over the competent *B. subtilis* wild type, the non-phosphorylatable *recAS2A* and phosphomimetic *recAS2D* mutant cells were transformed by the same amounts of the integrative plasmid pMUTIN2 containing different fragments of the *B. subtilis* chromosome. The efficiency of transformation was determined as the ratio of the erythromycine resistant colonies to the number of viable cells in competent cultures. The analysis was performed with three biological replicates; error bars represent the standard deviation. (B) Exponentially growing *B. subtilis* wild type and strains *recA* S2D and *recA* S2A were plated on LB-agar containing different concentrations of mitomycin C (indicated above the x-axis). The survival is expressed as the ratio of the colony-forming units from the cultures plated on LB + mitomycin C against those plated on LB. Presented results are mean values from three biological replicates, error bars represent standard deviations (C) The cells containing transcriptional fusion P_{dinR}*::lacZ* introduced in the wild type, *recA* S2D, *recA* S2A and Δ*yabT* background were grown to early exponential phase (OD_{600} 0.03) and SOS response was induced by 40 ng ml^{-1} of mitomycin C. β-galactosidase activity was plotted against time after induction. Black symbols represent the induced cultures and grey symbols uninduced. Error bars indicate standard deviations obtained from five independent experiments. (D) *In vitro* LexA cleavage by RecA wild-type (WT), RecA S2A and RecA S2D. Purified LexA protein was incubated with different forms of RecA for 4, 10 and 20 min. The proteins were separated by SDS-PAGE. Bands corresponding to RecA, intact LexA and LexA cleaved fragment are indicated by arrows. Quantification was performed from three independent experiments and the representative gel is shown. Columns 1 to 9 correspond to lanes 1 to 9 in the gel. The total amount of LexA was normalized to 1, and the percentage of LexA cleavage is shown.

Figure S5

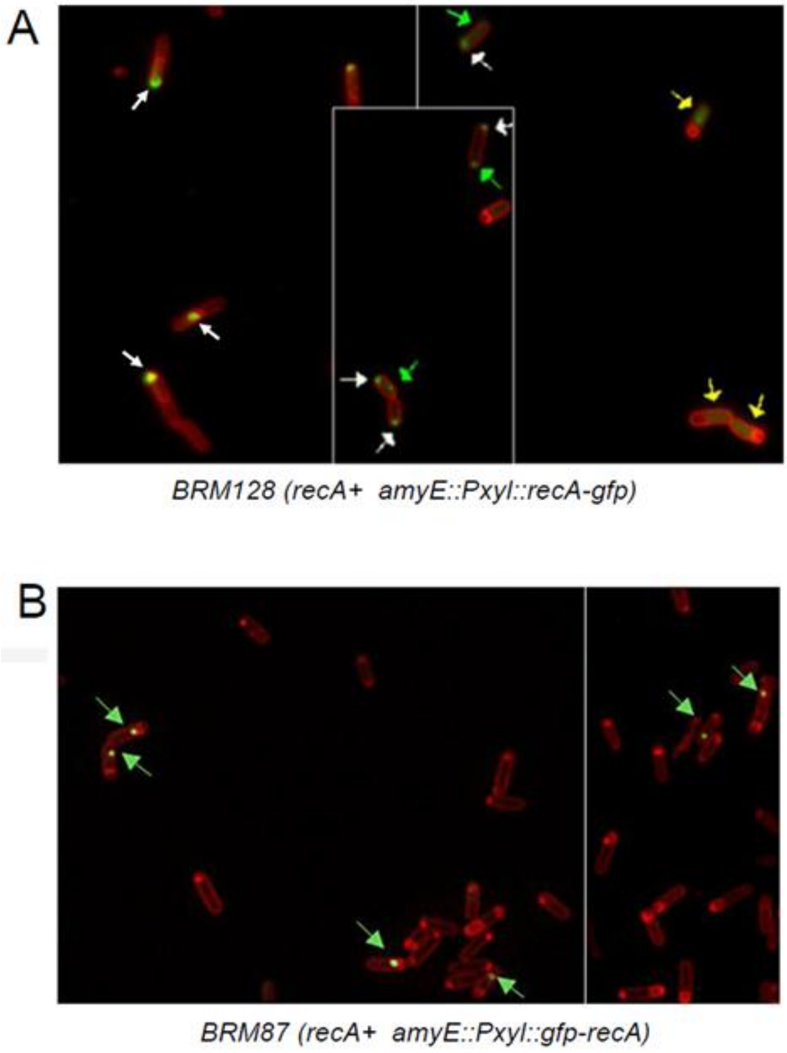

Figure S5. **RecA forms discrete foci during sporulation**. Examination of the C and N-terminal GFP-fusion to RecA. The GFP-fusions to RecA were expressed from a xylose-inducible promoter at the onset of sporulation (T0). Cells were mounted on 1.2% agarose pads and fluorescence was observed as described in Materiel and Methods. The overlay picture represents membrane-staining (FM4-64) in red and GFP is green. (A) Expression of the RecA-GFP from strain BMR 128 *(recA+ amyE::Pxyl::recA-gfp)*. The presence of polar aggregates or diffused RecA is indicated with white and yellow arrows, respectively. Discrete foci are indicated with green arrows. (B) Expression of the RecA-GFP form strain BMR 87 *(recA+ amyE::Pxyl:: gfp-recA)*. Discrete foci are indicated with green arrows. These foci are dynamic (see videos S1-S2). This construct has been retained for further analysis.

Figure S6

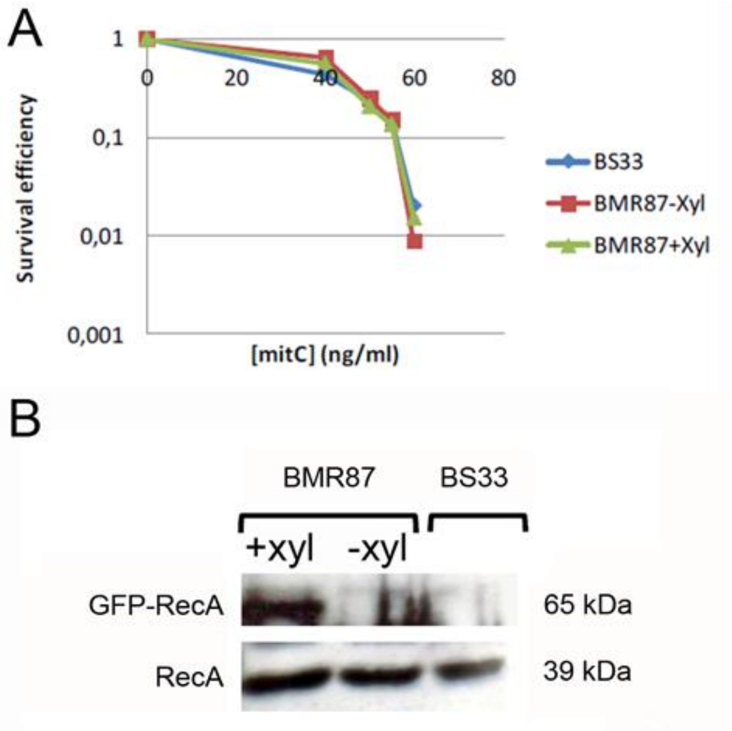

Figure S6. GFP-RecA expressed in the *recA*+ background. (A) The expression of *gfp-recA* from the xylose promoter in a *recA*+ background (BMR87) confers survival to increasing amount of mitomycin C similar to that of the wild-type (BS33). *Gfp-recA* fusion was induced by addition of 0.5% xylose, and cells were plated on LB-agar containing different concentrations of mitomycin C. Results were normalized with respect to 100% survival without mitomycin. (B) RecA (39 kDa) and GFP-RecA (65 kDa) protein levels were checked in protein extracts of wild type cells (BS33) and BMR87 (with and without the addition of xylose). Western detection was performed with the anti-RecA antibodies.

Figure S7

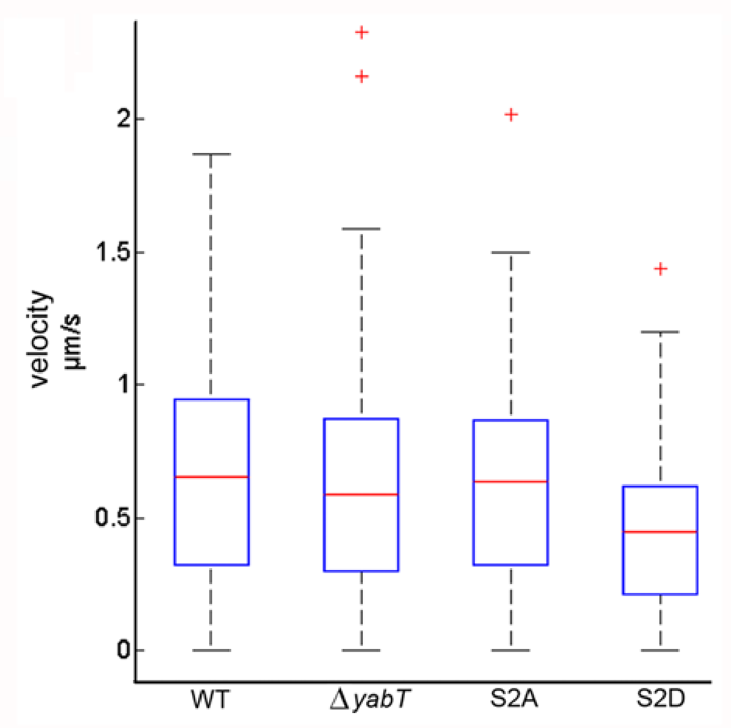

Figure S7. Statistical analysis of foci velocity. Box-plots of distributions of foci velocity. Strains are indicated on the x axis; S2A and S2D stand for *recA* S2A and *recA* S2D, respectively. Boxes correspond to the lower and upper quartiles, the median is indicated by a red line, extreme values by whiskers and outliers by red crosses. Distributions were determined from ~200 cells for each strain.

LEGENDS TO SUPPLEMENTARY VIDEOS

Movie S1. BMR87 fluorescence of GFP-RecA. Gfp-RecA fusions were expressed from a xylose-inducible promoter at the onset of sporulation (T0). Movies were mounted from 25 successive images plans, each exposed for 200 ms (total duration 5 s). Cells were observed at T3 corresponding to the expression peak for *yabT*. Membranes were stained with FM4–64. The movie exemplifies the typical motion of the wild type RecA foci.

Movie S2. RecA foci are mainly associated with DNA. Gfp-RecA fusion was induced at T0 and visualized as described above. *B. subtilis* cells expressing wild type GFP-RecA (green) were stained with DAPI to visualize the chromosome (pink) and FM4–64 to visualize the membranes at stage T3. This movie exemplifies the typical motion of a RecA focus in a chosen subset of cells of the wild type BMR87 strain.

Movie S3. RecA foci turn into threads upon treatment with MMC damaging agent. Fluorescence of GFP-RecA the wild type BMR87 strain after treatment with MMC. Cells were treated with 25 µg/ml of mitomycin at T1 and GFP-RecA fluorescence was recorded at T3. Movies were mounted from 25 successive images plans, each exposed for 200 ms (total duration 5 s). This movie exemplifies the typical motion of RecA in a chosen subset of cells.

Table S1. Localisation properties of tested GFP-tagged RecA fusions. Strains expressing a GFP-tagged RecA were carrying either a C-terminal fusion at the natural *recA* locus (LSA40), or ectopic C-terminal (LSA301, BMR126) and N-terminal (BMR87) GFP fusions in the presence of a wild type *recA* allele. Variants of GFP included GFPmut1, GFPmut2 and a GFPmut2 A206K variant described to promote GFP monomers (Simmons *et al*., 2007). Functionality refers to classical RecA function in survival to DNA damaging agent during exponential growth. Ectopic fusions were inducible by addition of xylose at the onset of sporulation to follow the localization of RecA, via the formation of a mixed nucleoprotein complex RecA/GFP-RecA. These results prompted us to monitor RecA localization during sporulation using BMR87 strain.

**strain**	**source**	**Type of *recA***					**Phenotype**		
		**untagged**	**GFP-tagged**				**Vegetative**	**Sporulation**	
		genomic copy	copy	expression	fusion	type	Functional*	Subcellar localisation	% cells with one focus
BMR 127	LAS40, (Simmons *et al*., 2007)	no	genomic locus	native	C-ter	*gfpmut2-A206K*	+/- §	no	no
BMR 128	LAS301, (Simmons *et al*., 2007)	yes	*lacA*	*Pxyl*	C-ter	*gfpmut2-A206K*	+	foci with inclusion body	>80%§§
BMR 126	*this work*	no	genomic locus	native	C-ter	*gfpmut1-A206K*	-	no	no
BMR 87	*this work*	yes	*amyE*	*Pxyl*	N-ter	*gfpmut1*	+	1 focus/cell	20%**

* survival to DNA damaging agent during exponential growth

** similar to what observed during exponential growth (this work, Miele *et al*., 2006)

§ functional at low dose of DNA-damaging agent at 30°C, but impaired at higher doses; not combinable with a *dnaX-fp* expressing stain (55)

§§ compared to <11% observed during exponential growth (Simmons *et al*., 2007)

Table S2. *B. subtilis* strains used in the study.

STRAINS			
Strain	Relevant genotype	Construction	Origin
BS33	*recA+*	TF8a x BEST5907 (P_r *::Neo; Nm*^{R})	this study
BMR76	*recAS2A*	See text for details	this study
BMR9	*recAS2D*	See text for details	this study
BMR25	Δ*yabT ::phleo*	See text for details; (Phl^R)	this study
BMR26	Δ*yabT ::Sp*	See text for details; (Sp^R)	this study
BMR87	*recA+* *amyE::*P_{xyl}*::gfp-recA*	BS33 x pSG1729*recA* (Sp^R)	this study
BMR81	*recAS2A amyE::*P_{xyl}*::gfp-recS2A*	BMR76 x pSG1729*recAS2A* (Sp^R)	this study
BMR16	*recAS2D amyE::*P_{xyl}*::gfp-recS2D*	BMR9 x pSG1729*recAS2D* (Sp^R)	this study
BMR86	*recAS2D* Δ*yabT ::phleo* *amyE::*P_{xyl}*::gfp-recAS2D*	BMR16 x BMR25 (Sp^R Phl^R)	this study
BMR88	Δ*yabT ::phleo* *amyE::*P_{xyl}*::gfp-recA*	BMR87 x BMR25 (Sp^R Phl^R)	this study
BMR154	Δ*yabT ::phleo* *amyE::*P_{xyl}*::gfp-yabT*	BMR25 x pSG1729*yabT* (Phl^R Sp^R)	this study
BMR155	Δ*yabT ::phleo* *amyE::*P_{xyl}*::gfp-yabT*Δ*TM*	BMR25 x pSG1729*yabT*Δ*TM* (Phl^R Sp^R)	this study
BMR157	*recAS2D* Δ*yabT ::Sp*	BS9 x BS26 (Sp^R)	this study
BMR6	*recA+ amyE::*P_{dinR}*::lacZ*	BS33 x JJS100	this study
BMR10	*recAS2D* *amyE::*P_{dinR}*::lacZ*	BMR9 x JJS100	this study
BMR78	*recAS2A* *amyE::*P_{dinR}*::lacZ*	BMR76 x JJS100	this study
BMR96	*yabT ::SPA*	BS33 x pMUTIN-SPA*yabT*	this study

Table S3. PCR primers used in this study. For mra primers, the nucleotides creating Ser2 to Ala2 mutation of the *recA* gene are bolded and underlined; the nucleotides complementary to the extremities of the insertion cassette are in small characters; and complementary sequences are marked in italics.

**PRIMERS (5'-3')**	
mra1	AGTCGGTTCAGAGTTGCTGCTTGG
mra3R	cgacctgcaggcatgcaagctGCCTGACGATCC**GC***CATTCTATTTTTTCCTCCTTTATGTTAC*C
mra2	TGTGTCTCTTCAGCTTGCTGCTG
mra3F	gagctcgaattcactggccgtc*GTAACATAAAGGAGGAAAAAATAGAATG***GC**GGATCGTCAGGCAGCC
mra4F	gagctcgaattcactggccgtc*GTAACATAAGGAGGAAAAAATAGAATG***GA**TGATCGTCAGGCAGCC
mra4R	cgacctgcaggcatgcaagctGCCTGACGATCA**TC***CATTCTATTTTTTCCTCCTTTATGTTAC*C
mra5	CATTCCCATGTTCTTCAGGCG
mra7F	gagctcgaattcactggccgtcGTGATCAGTGTTTTAGCGC
mra6	CGTATTCTCCAGGAGGAATG
mra7R	cgacctgcaggcatgcaagcTGGGAATATAGG CATCATCCG
RecA_wt forward pQE-30	CGGGATCCATGAGTGATCGTCAGGCAGCCTTAGATATGGC (*Bam*HI)
RecA_S2A forward pQE-30	CGCGGATCCATGGCTGATCGTCAGGCAGCCTTAGATATGGCTCT (S2A, *Bam*HI)
RecA_S2D forward pQE-30	CGCGGTCCATGGATGATCGTCAGGCAGCCTTAGATATGGCTCT (S2D, *Bam*HI)
RecA reverse pQE-30	AAAACTGCAGTTATTCTTCAAATTCGAGTTCTTCTTGTG (*Pst*I)
RecA_wt forward pQE-60	ACATGCCATGGGAAGTGATCGTCAGGCAGCCTTAGATATGGC (*Noc*I)
RecA_wt reverse pQE-60	GGAAGATCTTTCTTCAAATTCGAGTTCTTCTTGTGTCTC (*Bgl*II)
YabTt_forward	GAAGATCTATGATGAACGACGCTTTGACGAGTTTGGC (*Bgl*II)
YabTt_reverse	AAAACTGCAGTTAGATAAGCGTTGTTTCAAATAACCCC (*Pst*I)
YabT Δ1 forward	AAACTGCAGCCTAAGCTGTTCTTTGGGCTG (*Bgl*II)
YabT Δ2 forward	AAACTGCAGAGGCTGCGGTGATGCTTTTAT (*Bgl*II)

YabT K55D fwd	GAAGATCTATGGCCTTAGACGTGAGTGATGACAGC
YabT K55D rev	AAAACTGCAGTTAGTCATCACTCACGTCTAAGGCAACATG
YbdM_forward	CGGGATCCATGGCATTAAAACTTCTAAAAAAACTGC (*Bam*HI)
YbdM_reverse	AAAACTGCAGTTATGTGACCGATTGAATGGCCCG (*Pst*I)
PrkA forward	CGGGATCCATGGATATATTAAAGAAAATTGAAAAGTAC (*Bam*HI)
PrkA reverse	AAAACTGCAGTTATCGGTTCAGCAGGCTGCCG (*Pst*I)
PrkCt forward	GAAGATCTATGCTAATCGGCAAGCGGATCAGCGGGCG (*Bgl*II)
PrkCt reverse	AAAACTGCAGTTACAAAACCCACGGCCACTTTTTTCTTTTTGCCG (*Pst*I)
LexA_ forward	CGCGGATCCATGACGAAGCTATCAAAAAGG
LexA_ reverse	AAAACTGCAGTTAATGGACGGTTCTGAACAC
Gfp-yabT_forward	GAAGATCTATGGGTACCCTGCAGATGAGT
Gfp-yabT_ reverse	AAAACCCGGGTTAGATAAGCGTTGTTTCAAATAACCC
p*yabT*Apa	GCAGGGGCCCATGATGAACGACGCTTTGACG (*ApaI*)
p*yabT*Sal	ACGCGTCGACTCAGATAAAGAAAAAGATAATATAGGCG (*SalI*)
p*yabT* ΔTM	GTCGTGTCGACGGCTGCGGTGATG (*SalI*)
Universal	CGGAATTCTGCAGTTATTCTTCAAATTCGAGTTCTTCTTGTGTC
p*recA*wt	TTGGATCCATATGAGTGATCGTCAGGCAGCC
p*recA*S2A	TTGGATCCATATG**GC**TGATCGTCAGGCAGCC
p*recA*S2D	TTTGGATCCATATG**GA**TGATCGTCAGGCAGCC
*pyabT*Bam	GCAAGGATCCGCTGCATTGATGTGG
mra9	CGGAATTCTGCAGTTATTCTTCAAATTCGAGTTCTTCTTGTGTC (EcoRI, PstI)
p2HRwt	TTTGGATCCATATGAGTGATCGTCAGGCAGCC (BamHI)
p2HRS2A	TTTGGATCCATATGGCTGATCGTCAGGCAGCC (BamHI)
p2HRS2D	TTTGGATCCATATGGATGATCGTCAGGCAGCC (BamHI)
p2HyabT-F	GATTCCCGGGATGATGAACGACGCTTTGACGAG (SmaI)
p2HyabT-R	CGCGTCGACTCAGATTAAGAAAAAGATAATATAGGCG (SalI)
